# Neuroinflammation: a critical bridge linking peripheral pathology and age-related degeneration in myasthenia gravis

**DOI:** 10.3389/fmed.2026.1746161

**Published:** 2026-05-01

**Authors:** Fan-yu Liu, Yan-peng Huang, Zhao-qing Li, Xu Li, Jing-sheng Zhang, Le Guan, Wen-jun Qiao

**Affiliations:** 1 Liaoning University of Traditional Chinese Medicine, Shenyang, China; 2 Affiliated Hospital of Liaoning University of Traditional Chinese Medicine, Shenyang, China

**Keywords:** age-stratified therapy, myasthenia gravis, immunosenescence, microglia, mitochondrial dysfunction, neurodegeneration, neuroinflammation

## Abstract

Myasthenia gravis (MG) has traditionally been conceptualized as a peripheral autoimmune disorder primarily mediated by autoantibodies targeting the neuromuscular junction. However, this classical paradigm fails to adequately explain the prevalent central nervous system (CNS) manifestations in patients, including profound fatigue and cognitive impairment. Emerging evidence indicates that neuroinflammation plays a pivotal role in bridging peripheral pathology and central symptoms. Systemic inflammatory mediators can breach the compromised blood-brain barrier (BBB) or activate CNS-resident microglia and astrocytes via neuroimmune pathways, thereby initiating neuroinflammatory cascades. Once activated, these glial cells release pro-inflammatory cytokines and reactive oxygen species (ROS), which impair neuronal energy metabolism, synaptic plasticity, and neurotransmitter homeostasis, directly contributing to central symptomatology. Critically, neuroinflammation serves as a key mechanistic bridge linking the peripheral autoimmune pathology of MG with age-related neurodegenerative changes. With advancing age, immunosenescence manifests as diminished T-cell repertoire diversity, impaired regulatory T-cell function, and chronic low-grade inflammation (inflammaging), which not only increases susceptibility to MG but also provides a permissive environment for the initiation and perpetuation of neuroinflammation. Concurrently, age-related degenerative alterations at the neuromuscular junction—including reduced acetylcholine receptor (AChR) density and mitochondrial dysfunction—decrease the safety margin of neuromuscular transmission, rendering elderly patients more vulnerable to autoantibody-mediated attack. A vicious cycle emerges among neuroinflammation, mitochondrial dysfunction, and oxidative stress, which synergistically accelerate neuronal damage and apoptosis. Consequently, the clinical phenotype, therapeutic response, and prognosis of MG demonstrate marked age-dependency. Late-onset MG patients typically experience more severe disease courses and poorer outcomes, attributable in part to the compounding effects of immunosenescence, underlying neurodegeneration, and neuroinflammation. Elucidating the central role of neuroinflammation and its intricate interactions with age-related pathological processes holds significant theoretical and clinical implications for developing novel neuroprotective strategies targeting CNS symptoms in MG and achieving personalized, precision medicine tailored to patients across different age groups.

## 1 Introduction

Myasthenia gravis (MG) is an antibody-mediated autoimmune disorder characterized by impaired neuromuscular junction transmission, manifesting as fluctuating muscle weakness (1). Traditionally, MG has been conceptualized as a peripheral disease, primarily driven by immune-mediated attacks on the postsynaptic membrane through acetylcholine receptor antibodies (AChR-Ab) or muscle-specific kinase antibodies (MuSK-Ab), resulting in postsynaptic dysfunction (2). However, this “peripheral pathology” model fails to adequately explain the prevalent central nervous system (CNS) symptoms observed in patients, such as fatigue and cognitive impairment. Recent studies have demonstrated significantly elevated levels of pro-inflammatory cytokines, particularly interleukin-6 (IL-6), in the serum of MG patients (3). Notably, the 2024 study by Yang et al. (4) revealed that infection and chronic diseases can activate a brain-muscle signaling axis, with centrally-derived IL-6 directly regulating muscle function, suggesting a critical role for bidirectional peripheral-central immune communication in MG pathogenesis. With accelerating global population aging, the proportion of late-onset MG (age of onset ≥50 years) continues to increase (5). Age-related factors, including immunosenescence, neuromuscular junction degeneration, and neuroinflammation, play important roles in MG pathogenesis.

Neuroinflammation represents a complex immune response of the central nervous system to pathological stimuli. In MG, systemic inflammatory mediators can penetrate the compromised blood-brain barrier (BBB) to enter the central nervous system, activating microglia to release pro-inflammatory cytokines such as tumor necrosis factor-α (TNF-α) and interleukin-1β (IL-1β), thereby initiating a neuroinflammatory cascade (6). Activated microglia further induce astrocyte transformation toward a neurotoxic phenotype, with these two cell types forming a positive feedback loop that significantly amplifies the inflammatory response (7). This glia-mediated neuroinflammation ultimately leads to neuronal dysfunction and impaired synaptic plasticity by damaging neuronal energy metabolism, disrupting neurotransmitter homeostasis, and inducing oxidative stress, thereby mediating the central symptoms of MG (8).

Based on the above pathological observations, we propose the following testable mechanistic hypothesis: persistently elevated peripheral pro-inflammatory mediators in MG patients, particularly IFN-γ and IL-17, mediate peripheral-central immune crosstalk through a dual-pathway mechanism.

First, the paracellular pathway: IFN-γ and TNF-α activate the NF-κB signaling pathway, leading to downregulation of tight junction proteins (claudin-5, occludin, and ZO-1) in cerebrovascular endothelial cells and triggering caspase-3/9-mediated tight junction remodeling. These molecular events collectively increase blood-brain barrier (BBB) paracellular permeability, thereby permitting passive diffusion of peripheral inflammatory mediators and autoantibodies into the central nervous system (CNS) (9, 10). *In vitro* studies have demonstrated that combined treatment with TNF-α and IFN-γ significantly reduces transendothelial electrical resistance in brain endothelial cells and induces redistribution of occludin and ZO-1 from intercellular junctions to the cytoplasm (10).

Second, the transcellular active transport pathway: IL-17 and IL-22 act directly on cerebrovascular endothelial cells expressing their cognate receptors, disrupting tight junction integrity and upregulating expression of adhesion molecules (ICAM-1 and VCAM-1) on the endothelial cell surface, thereby facilitating Th17 cell transmigration across the BBB (11). Studies have confirmed that in active multiple sclerosis (MS) lesions, BBB endothelial cells exhibit elevated expression of IL-17 and IL-22 receptors. IL-17 treatment induces downregulation of occludin and ZO-1 expression and enhances secretion of the chemokine CCL2, further promoting transendothelial migration of CD4+ T cells (11, 12). Moreover, upregulation of ICAM-1 and VCAM-1 on brain endothelial cells represents a critical molecular event mediating adhesion and transmigration of Th1 and Th17 cells across the BBB (13).

### 1.1 Experimental approaches to test the hypothesis

This hypothesis could be experimentally tested through the following approaches: (i) analysis of correlations between peripheral blood cytokine profiles (IFN-γ, IL-17, IL-6, and TNF-α) and cerebrospinal fluid (CSF) inflammatory markers (such as CXCL13 and neurofilament light chain [NfL]) in MG patients; (ii) quantitative assessment of BBB permeability using dynamic contrast-enhanced magnetic resonance imaging (DCE-MRI) to measure the volume transfer constant (Ktrans) (14); and (iii) evaluation of associations between peripheral blood Th17 cell proportions and CNS symptom severity. Notably, previous studies have provided preliminary evidence of CSF immune activation in MG patients, with approximately 50% of patients demonstrating elevated lymphocytic reactivity in CSF (15), which may offer indirect support for the peripheral-central inflammatory transmission hypothesis, though further investigation is needed to establish causality.

Immunosenescence constitutes a crucial biological foundation for the high incidence of late-onset MG. With advancing age, T cell repertoire diversity diminishes, regulatory T cell function declines, and chronic inflammatory states (inflammaging) intensify, leading to immune homeostasis disruption and increased susceptibility to autoimmunity (16). Concurrently, age-related degenerative changes in the neuromuscular junction reduce the safety factor of neurotransmission. Presynaptic mitochondrial dysfunction decreases adenosine triphosphate (ATP) production, affecting acetylcholine synthesis and release, while postsynaptic acetylcholine receptor density declines and receptor clustering function becomes impaired. These changes collectively render the elderly population more vulnerable to autoantibody attacks (17). Age-related chronic low-grade inflammation serves as a core driving force of neurodegenerative pathology. Sustained activation of microglia and astrocytes releases excessive pro-inflammatory factors that directly damage neurons (18). Chronic neuroinflammation and oxidative stress constitute a mutually reinforcing dynamic process: activated glial cells generate abundant reactive oxygen species (ROS) through nicotinamide adenine dinucleotide phosphate (NADPH) oxidase, while ROS activates pro-inflammatory signaling pathways such as nuclear factor-κB (NF-κB), further amplifying inflammation (19). This self-reinforcing vicious cycle accelerates pathological protein deposition and causes widespread cellular damage.

Mitochondrial dysfunction occupies a central position in the aforementioned processes. In MG patients, mitochondrial damage results in insufficient ATP synthesis, causing energy supply dysfunction at the neuromuscular junction and exacerbating muscle weakness and fatigue symptoms (20). Dysfunctional mitochondria become a major source of ROS, triggering severe oxidative stress. Mitochondrial membrane potential impairment leads to calcium homeostasis dysregulation, and ROS accumulation combined with calcium overload initiates apoptotic programs through the mitochondrial pathway, including mitochondrial permeability transition pore opening and cytochrome c release, ultimately resulting in motor nerve terminal degeneration and muscle fiber loss (21).

Complex bidirectional regulatory relationships exist between neuroinflammation and age-related degenerative pathology. Damage-associated molecular patterns (DAMPs) released by injured neurons, such as pathological proteins, extracellular ATP, and high mobility group box 1 protein (HMGB1), can activate pattern recognition receptors on the glial cell surface, triggering neuroinflammation (22). Inflammatory mediators cause further damage to adjacent neurons by inducing oxidative stress, disrupting calcium homeostasis, and impairing mitochondrial function. This forms a sustained feedback loop: inflammation promotes cellular damage and apoptosis, newly apoptotic neurons release more DAMPs, further activating glial cells, ultimately leading to persistent neuronal loss (18, 22).

In conclusion, neuroinflammation serves as a critical bridge connecting peripheral pathology and central symptoms in MG, while age-related factors significantly influence MG onset and progression through immunosenescence, neuromuscular junction degeneration, and activation of the neuroinflammation-oxidative stress-mitochondrial dysfunction axis. Therefore, a comprehensive understanding of these interactive mechanisms holds important scientific significance and clinical value for elucidating clinical heterogeneity across different age groups, developing novel therapeutic strategies targeting neuroinflammation and mitochondrial function, and predicting disease progression. To achieve the above objectives, we conducted a comprehensive systematic literature search across multiple databases (PubMed, Web of Science, Scopus, and Cochrane Library) from January 2000 to May 2025. Detailed search strategies, inclusion/exclusion criteria, and screening procedures are provided in Supplementary material S1.

## 2 Immunological basis of myasthenia gravis

### 2.1 Autoantibody abnormalities

#### 2.1.1 Anti-acetylcholine receptor antibodies

**Figure 1:**
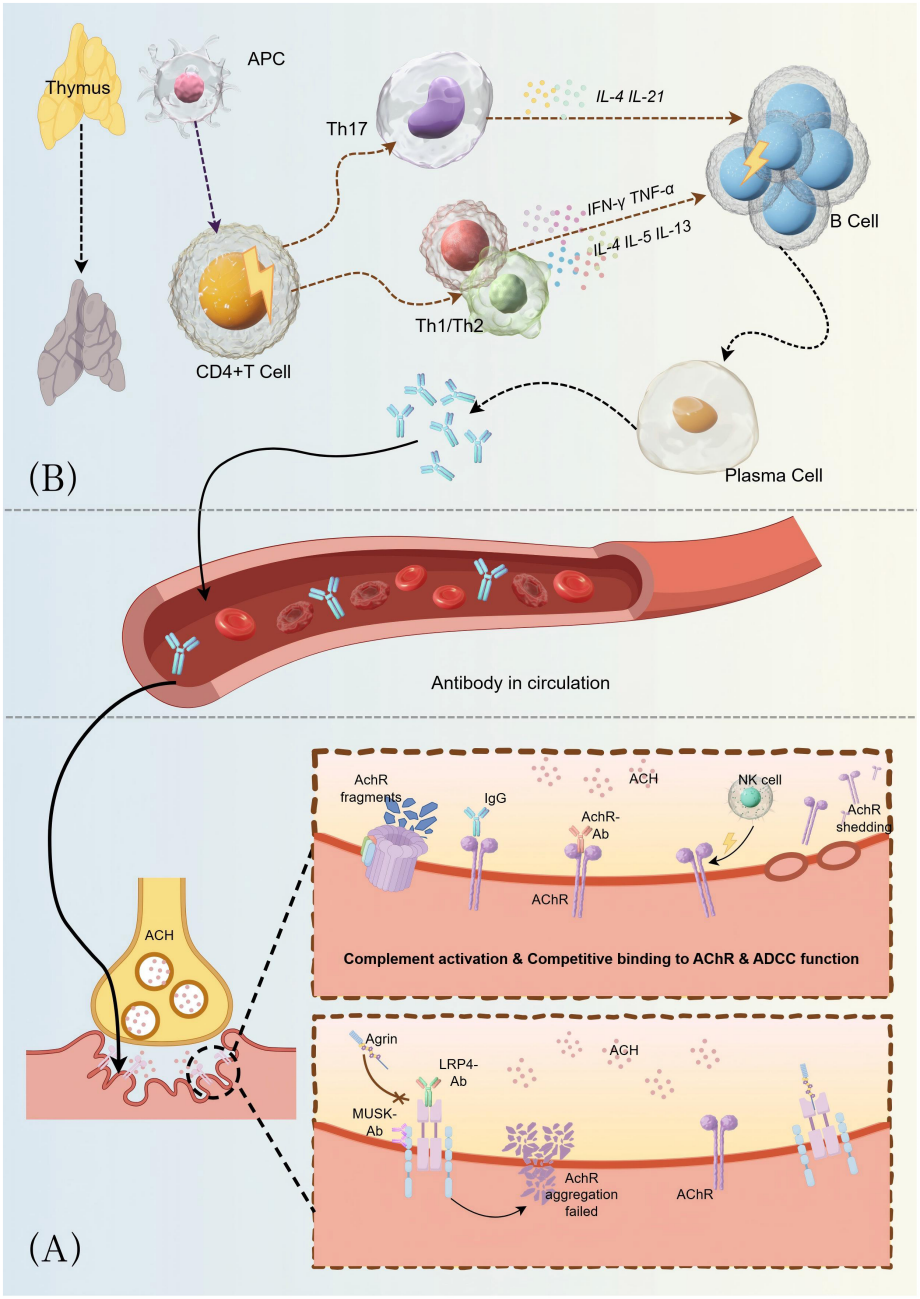
Schematic representation of autoantibody-mediated impairment of neuromuscular transmission in myasthenia gravis. **(A)** Normal neuromuscular junction transmission: upon arrival of a nerve impulse at the motor nerve terminal, voltage-gated calcium channels open, triggering calcium influx that promotes fusion of acetylcholine (ACh)-containing synaptic vesicles with the presynaptic membrane. ACh is subsequently released into the synaptic cleft via exocytosis and binds to nicotinic acetylcholine receptors (AChRs) densely clustered on the postsynaptic membrane. This binding initiates the generation of endplate potentials (EPPs); when the EPP amplitude reaches threshold, it triggers an action potential in the muscle fiber, culminating in muscle contraction. Released ACh is rapidly hydrolyzed by acetylcholinesterase (AChE) localized in the synaptic cleft, ensuring precise temporal control of neuromuscular transmission. **(B)** Autoimmune attack in myasthenia gravis: (1) Initiation of autoimmune response: within lymphoid organs, particularly the thymus, autoreactive CD4+ T cells undergo aberrant activation and provide cognate help to B cells, promoting their differentiation into antibody-secreting plasma cells. These plasma cells produce pathogenic autoantibodies directed against neuromuscular junction (NMJ) components, predominantly AChRs, but also muscle-specific kinase (MuSK), lipoprotein receptor-related protein 4 (LRP4), and other postsynaptic proteins. (2) Antibody-mediated pathological mechanisms: (i) Functional blockade: anti-AChR antibodies competitively inhibit ACh binding to its receptor by occupying or sterically hindering the ligand-binding site, thereby reducing the efficiency of neuromuscular transmission. Anti-MuSK antibodies disrupt the agrin-LRP4-MuSK signaling pathway, which is essential for AChR clustering and maintenance at the postsynaptic membrane, leading to dispersal and reduced density of AChRs. (ii) Complement-mediated cytotoxicity**:** binding of IgG autoantibodies (particularly IgG1 and IgG3 subclasses) to AChRs activates the classical complement cascade, culminating in formation of the membrane attack complex (MAC, C5b-9). MAC insertion into the postsynaptic membrane causes focal lysis, structural disintegration of junctional folds, and accelerated internalization and degradation of AChRs. (iii) Antibody-dependent cell-mediated cytotoxicity (ADCC): Natural killer (NK) cells and other effector cells recognize the Fc portion of receptor-bound antibodies via Fc receptors, triggering release of cytotoxic granules containing perforin and granzymes. This process induces localized destruction of the postsynaptic membrane and shedding of AChR-containing membrane fragments. (iv) Structural and functional disruption of the postsynaptic membrane: the convergence of these pathological mechanisms results in a dramatic reduction in functional AChR density (often >70% loss), simplification and fragmentation of postsynaptic junctional folds, and compromised safety margin of neuromuscular transmission. Consequently, the diminished population of available AChRs cannot generate EPPs of sufficient amplitude to reliably reach the threshold for action potential initiation in muscle fibers. This transmission failure manifests clinically as muscle weakness, abnormal fatigability upon repetitive stimulation, and the characteristic fluctuating symptomatology of myasthenia gravis. Created with Figdraw (www.figdraw.com).

Figure 1 illustrated the Immunological Basis of MG. Anti-acetylcholine receptor antibodies (AChR-Ab) are the predominant pathogenic autoantibodies in myasthenia gravis (MG), mediating neuromuscular transmission failure through three mechanisms. First, functional blockade occurs when antibodies directly occupy acetylcholine binding sites on the postsynaptic receptor, preventing neurotransmitter-receptor interaction and inhibiting the depolarization cascade essential for muscle contraction. Second, complement-mediated cytotoxic injury ensues when antibody-antigen immune complexes activate the classical complement pathway, forming membrane attack complexes (MAC) that cause focal lysis and structural disruption of the postsynaptic membrane. This complement-dependent mechanism represents the primary pathogenic cascade responsible for ultrastructural damage at the neuromuscular junction in AChR-positive MG. Third, antigenic modulation accelerates AChR internalization and lysosomal degradation through antibody-induced receptor cross-linking, reducing functional receptor density at the neuromuscular junction by approximately 70%−90% (23). This multifaceted antibody-mediated assault significantly compromises neuromuscular transmission, manifesting as the fluctuating muscle weakness and fatigability characteristic of MG.

#### 2.1.2 Anti-muscle-specific kinase antibodies

Myasthenia gravis mediated by anti-muscle-specific kinase antibodies (MuSK-Ab) exhibits distinct pathogenic mechanisms compared to AChR-MG. MuSK-Ab predominantly belong to the immunoglobulin G4 (IgG4) subclass, which possesses minimal complement-activating capacity (24). Rather than triggering complement-mediated membrane destruction, these antibodies disrupt the interaction between MuSK and its ligand, low-density lipoprotein receptor-related protein 4 (LRP4), blocking the agrin-LRP4-MuSK signaling cascade essential for AChR clustering and stabilization at the postsynaptic membrane (24). MuSK-Ab-mediated pathology primarily manifests as dispersal and loss of AChR aggregates rather than structural membrane damage, with preservation of postsynaptic membrane integrity despite functional impairment. These distinct mechanisms account for the characteristic clinical features of MuSK-MG, including marked predilection for bulbar, cervical, and respiratory muscle involvement, and notably poor response to cholinesterase inhibitors (25). The IgG4-predominant antibody profile has important therapeutic implications, as IgG4 antibodies exhibit reduced susceptibility to conventional immunosuppressive regimens targeting complement-fixing antibody subclasses.

### 2.2 Cellular immune dysregulation

CD^4+^ T cell abnormalities constitute a cardinal feature of MG pathogenesis, orchestrating the dysregulated immune response. T helper 1 (Th1) cells secrete interferon-gamma (IFN-γ) and tumor necrosis factor-alpha (TNF-α), enhancing B cell antibody secretion and upregulating MHC class II expression on muscle cells, thereby augmenting antigen presentation. Th2 cells drive B cell activation and IgG-class autoantibody production through interleukin-4 (IL-4), IL-5, and IL-13 secretion (26). This Th1/Th2 imbalance forms the immunological foundation for humoral immunity predominance in MG. Follicular helper T (Tfh) cells, which facilitate B cell maturation and antibody class switching within germinal centers, are significantly elevated in peripheral blood and thymic tissue of MG patients, correlating with disease severity and autoantibody titers (27).

Regulatory T cells (Tregs) in MG patients exhibit reduced numbers, decreased Foxp3 expression, and impaired suppressive function, unable to effectively constrain autoreactive T and B cell populations. Conversely, Th17 cells demonstrate pathological overactivation with excessive IL-17, IL-21, and IL-22 secretion, recruiting inflammatory cells to the neuromuscular junction and promoting B cell differentiation into antibody-secreting plasma cells (28). The elevated Th17/Treg ratio has emerged as a valuable biomarker for assessing disease severity and monitoring therapeutic response (28).

B cell activation in MG occurs through coordinated costimulatory signals from helper T cells and stimulation by IL-21 and IL-6, driving differentiation into plasma cells under Blimp-1 transcriptional control (29). The thymus and peripheral lymphoid tissues harbor ectopic germinal centers providing specialized microenvironments for B cell activation, somatic hypermutation, and affinity maturation, generating high-affinity anti-AChR antibody-producing clones. Plasma cells comprise short-lived populations that rapidly secrete antibodies but undergo apoptosis within days to weeks, and long-lived populations that persist in bone marrow survival niches for months to years (29). Long-lived plasma cells, being terminally differentiated with downregulated B cell surface markers including CD20, remain resistant to B cell-depleting therapies such as rituximab, necessitating approaches targeting the plasma cell compartment for sustained therapeutic benefit.

### 2.3 Pathophysiological role of the thymus in myasthenia gravis

The thymus occupies a pivotal position in MG pathogenesis. Approximately 60%−70% of MG patients exhibit thymic hyperplasia characterized by ectopic germinal center-like structures within the thymic medulla (30). These aberrant lymphoid structures, enriched with activated B cells, plasma cells, and follicular dendritic cells, transform the thymus into an ectopic lymphoid organ sustaining anti-AChR antibody production independently of peripheral lymphoid tissues (30). An additional 10%−15% of patients present with thymoma, disrupting thymic architecture and impairing T cell selection processes, resulting in extensive tolerance breakdown and often refractory disease (31).

Under physiological conditions, medullary thymic epithelial cells (mTECs) express diverse tissue-restricted antigens, including AChR subunits, under AIRE gene regulation. This promiscuous gene expression enables negative selection of autoreactive T cells, establishing central tolerance (32). However, in some MG patients, reduced AIRE expression or loss-of-function mutations compromise this mechanism, allowing autoreactive T cells to escape thymic deletion (32). The aberrant expression of neuromuscular antigens within the pathological thymus provides a persistent antigen source for activating autoreactive T cells, while ectopic germinal centers support B cell affinity maturation (33).

Thymectomy eliminates ectopic germinal centers, interrupting sustained autoantibody production, and depletes the thymic antigen reservoir perpetuating autoreactive T cell expansion (34). The MGTX trial demonstrated that post-thymectomy patients experience sustained reduction in anti-AChR antibody titers, decreased autoreactive T cell frequencies, and improved clinical outcomes with reduced immunosuppressive requirements. Therapeutic benefit appears most pronounced in younger patients (particularly under 50 years) and those with documented thymic hyperplasia (34).

### 2.4 Breakdown of immune tolerance

Immune tolerance breakdown in MG involves defects in both central and peripheral mechanisms. At the central level, AIRE dysfunction impairs tissue-restricted antigen expression by mTECs, preventing adequate thymic presentation during T cell development (35). Human leukocyte antigen (HLA) polymorphisms, particularly HLA-B8, DR3, and DQ2 haplotypes, confer increased MG susceptibility through altered self-antigen presentation and processing (35).

Peripherally, numerical reduction and functional deficiency of Tregs constitute the most significant tolerance deficit, severely compromising suppression of autoreactive effector cells (26). Dysfunction of immune checkpoint molecules, including CTLA-4 and PD-1, lowers the activation threshold for T cell responses to self-antigens (36).

The clinical relevance of checkpoint dysfunction is illustrated by immune checkpoint inhibitor-associated MG in cancer patients receiving anti-PD-1 (pembrolizumab, nivolumab) or anti-CTLA-4 (ipilimumab) therapy. These agents can trigger severe MG, often presenting with myasthenic crisis (37). Beyond pharmacological triggers, physiological stressors including stress and pregnancy may precipitate disease onset. Recent investigations implicate gut microbiome alterations in MG pathogenesis, suggesting dysbiosis contributes to systemic immune dysregulation through effects on mucosal immunity (38). These diverse factors underscore the complex interplay between genetic predisposition, immune dysregulation, and environmental triggers in MG pathogenesis (38).

## 3 The role of neuroinflammation in myasthenia gravis

**Figure 2:**
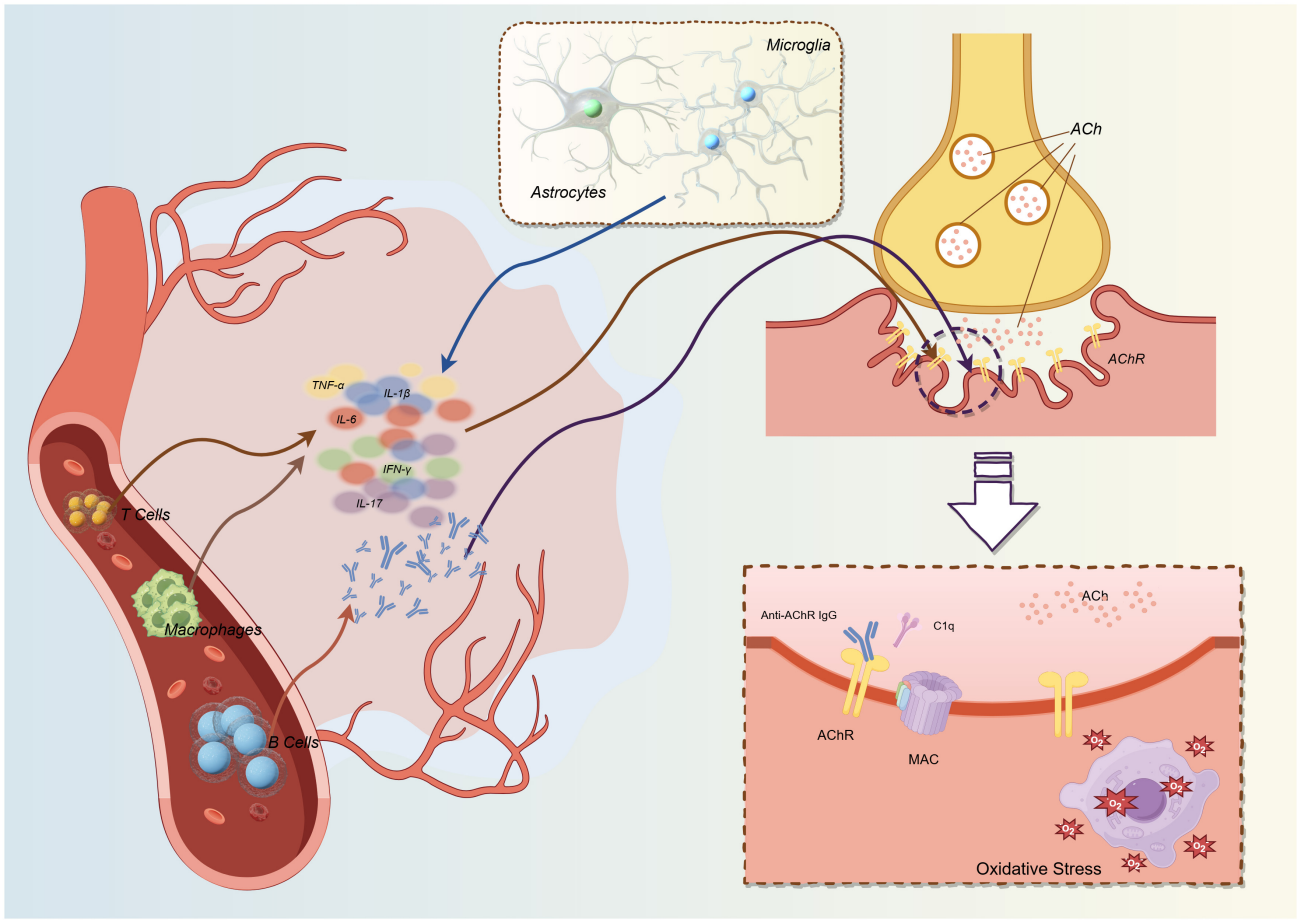
Neuroinflammation-mediated damage mechanisms at the neuromuscular junction in myasthenia gravis. The pathological progression of myasthenia gravis initiates with aberrant infiltration of pro-inflammatory immune cells. Effector T cells, autoreactive B cells, and activated macrophages from the peripheral circulation breach the blood-nerve barrier and migrate to the neuromuscular junction. Concurrently, resident microglia and astrocytes become activated and adopt a pro-inflammatory phenotype. These immune cells collectively secrete abundant pro-inflammatory cytokines [including tumor necrosis factor-α (TNF-α), interleukin-1β (IL-1β), interleukin-6 (IL-6), interferon-γ (IFN-γ), and interleukin-17 (IL-17)], establishing a sustained, self-amplifying inflammatory network within the neuromuscular junction microenvironment that specifically targets postsynaptic membrane structures. This dysregulated inflammatory cascade induces progressive postsynaptic membrane destruction through three major synergistic pathological mechanisms: (1) Antibody-dependent complement-mediated cytolysis: autoantibodies (primarily anti-acetylcholine receptor [anti-AChR] antibodies) specifically bind to AChRs on the postsynaptic membrane, triggering activation of the classical complement pathway. The complement cascade culminates in assembly and insertion of the membrane attack complex (MAC, C5b-9), forming transmembrane pores that cause physical membrane disruption, ionic homeostasis imbalance, and cytoskeletal disintegration. (2) Direct cytokine-mediated toxicity: pathologically elevated concentrations of pro-inflammatory cytokines (particularly TNF-α and IL-1β) directly initiate apoptotic programs in postsynaptic membrane cells through their cognate receptor signaling pathways. These cytokines also downregulate AChR expression and clustering, accelerate receptor internalization and degradation, and suppress synthesis and trafficking of new receptors, thereby systematically compromising neuromuscular transmission efficiency. (3) Oxidative stress and mitochondrial dysfunction: excessive reactive oxygen species (ROS) and reactive nitrogen species (RNS) generated in the inflammatory microenvironment, along with intracellular ROS released from damaged mitochondria, collectively cause severe oxidative stress injury. This results in lipid peroxidation, protein oxidative modification, and DNA damage, further exacerbating degenerative changes in postsynaptic structures. The synergistic effects of these pathological mechanisms ultimately lead to: (i) dramatic reduction in functional AChR density (to < 30% of normal levels); (ii) simplification and loss of characteristic postsynaptic membrane folds; (iii) significant reduction in postsynaptic membrane surface area; and (iv) decreased neuromuscular transmission safety factor, manifesting clinically as muscle weakness and fatigability. This figure systematically illustrates the complete pathological cascade from immune cell infiltration to synaptic structural destruction. Created with Figdraw (www.figdraw.com).

As shown in Figure 2, although the pathological core of myasthenia gravis (MG) is localized to the neuromuscular junction within the peripheral nervous system, the disease is fundamentally a systemic autoimmune disorder characterized by widespread immune dysregulation. The abundant inflammatory mediators generated by systemic immune perturbations can exert direct effects at the neuromuscular junction, while simultaneously influencing the central nervous system (CNS) through mechanisms such as blood-brain barrier (BBB) disruption or activation of neuroimmune axes, thereby triggering central neuroinflammation (3, 4). Conversely, inflammatory states within the CNS may reciprocally modulate peripheral immune responses, establishing a bidirectional communication network. This dynamic bidirectional crosstalk positions neuroinflammation as a critical bridge connecting the classical peripheral manifestations of MG with its central neurological features, including profound fatigue and cognitive alterations. Consequently, investigating the activation of microglia and astrocytes, along with their mediated inflammatory cascades, holds substantial significance for comprehensively understanding the pathophysiology and clinical presentations of MG. This chapter systematically examines the multifaceted roles of neuroinflammation in MG, encompassing the expression and regulation of inflammatory mediators, the involvement of glial cells, and the complex interactions between neuroinflammation and infiltrating immune cells.

### 3.1 Expression and regulation of inflammatory mediators

In patients with MG, aberrant expression of proinflammatory cytokines is recognized as an integral component of the disease's pathological mechanisms, with interleukin-6 (IL-6) and tumor necrosis factor-alpha (TNF-α) playing particularly pivotal roles in disease pathogenesis. Accumulating evidence demonstrates that serum IL-6 levels are significantly elevated in MG patients, exhibiting strong correlations with disease activity and severity indices (3). Beyond its participation in localized inflammatory responses, IL-6 exerts pleiotropic effects through activation of the Janus kinase-signal transducer and activator of transcription (JAK-STAT) signaling pathway, thereby promoting B cell differentiation and autoantibody production. Furthermore, IL-6-driven systemic inflammatory responses may directly contribute to the exacerbation of muscle fatigability, a cardinal clinical feature of MG (3, 4). TNF-α similarly occupies a central position in MG pathophysiology, not only serving as a key mediator of inflammatory cascades but also synergizing with IL-6 to compromise the structural integrity of the neuromuscular junction and impair acetylcholine receptor function, ultimately culminating in neurotransmission failure (39, 40). The synergistic interactions between these cytokines amplify their individual pathogenic effects, creating a self-perpetuating inflammatory milieu that sustains disease activity.

The pathogenic effects of these cytokines are largely mediated through activation of complex downstream signaling pathway networks that orchestrate inflammatory responses and cellular dysfunction. The release of IL-6, TNF-α, and other proinflammatory cytokines triggers activation of key intracellular signaling cascades, including nuclear factor-kappa B (NF-κB) and mitogen-activated protein kinase (MAPK) pathways (39, 40). NF-κB activation, in particular, not only promotes the transcriptional upregulation of numerous inflammatory mediators, thereby amplifying the inflammatory response, but may also directly induce neuronal dysfunction through alterations in gene expression programs (39, 40). Additionally, activation of the NOD-like receptor protein 3 (NLRP3) inflammasome, a critical component of the innate immune system, has been identified as a key proinflammatory process in MG pathogenesis. Recent investigations have revealed that serum levels of pentraxin 3 (PTX3) are elevated in MG patients, capable of promoting NLRP3 inflammasome activation and subsequent pyroptotic cell death (41, 42). This inflammasome-mediated cellular response results in the proteolytic maturation and release of IL-1β and IL-18, potent proinflammatory cytokines that further intensify inflammatory cascades and compromise both neurological function and the maintenance of muscle strength (41, 42). Moreover, sustained elevation of inflammatory mediators, particularly chronic IL-6 exposure, may compromise BBB integrity through disruption of tight junction proteins and endothelial cell dysfunction, thereby facilitating the infiltration of peripheral immune cells into the CNS and exacerbating local neuroinflammatory processes (43). This breach of the BBB represents a critical mechanistic link between peripheral immune dysregulation and central neurological manifestations in MG.

### 3.2 Participation of microglia and astrocytes in neuroinflammation

In the pathological mechanisms underlying MG, neuroinflammatory responses within the CNS have garnered increasing attention, with the intricate interplay between microglia and astrocytes emerging as a particularly critical component of disease pathophysiology. As the resident immune cells of the CNS, microglia serve as the primary sentinels and responders to injury signals and pathological stimuli within the nervous system (44). Accumulating evidence indicates that in autoimmune diseases such as MG, peripheral inflammatory mediators can traverse a compromised BBB to enter the CNS, where they activate microglia and trigger the release of proinflammatory factors including TNF-α and IL-1β, thereby participating in local immune modulation (44). While this microglial response may initially serve a protective function aimed at maintaining neuronal homeostasis and clearing potentially harmful stimuli, persistent or excessive activation transitions into a maladaptive state that perpetuates neuroinflammation. Such sustained microglial activation can directly or indirectly induce neuronal injury and functional impairment through the continuous production of neurotoxic mediators, reactive oxygen species, and proteolytic enzymes, ultimately contributing to the neurological symptoms observed in MG patients.

#### 3.2.1 Microglial polarization states

In MG and related autoimmune models, microglia exhibit pronounced polarization toward the M1 pro-inflammatory phenotype, characterized by elevated expression of CD86, inducible nitric oxide synthase (iNOS), and major histocompatibility complex class II (MHC-II), accompanied by substantial secretion of TNF-α, IL-1β, IL-6, and reactive oxygen species (ROS) (44–46). In contrast, the neuroprotective M2 phenotype [characterized by markers including CD206, arginase-1 (Arg-1), and IL-10] is markedly diminished in chronic inflammatory environments (46, 47). This M1/M2 imbalance creates a persistent neurotoxic microenvironment.

Furthermore, the complex bidirectional interactions between microglia and astrocytes constitute an indispensable component of neuroinflammatory pathology, with profound implications for disease progression and neurological dysfunction. Astrocytes play fundamental roles in maintaining neuronal microenvironmental homeostasis through diverse mechanisms, including regulation of synaptic transmission, maintenance of ionic and neurotransmitter balance, provision of metabolic support to neurons, and modulation of BBB integrity (48). Activated microglia can induce astrocytic transformation toward a reactive phenotype through the release of cytokines such as TNF-α and IL-1β, along with various chemokines and other signaling molecules (48, 49). This reactive astrogliosis is characterized by increased expression of glial fibrillary acidic protein (GFAP), morphological hypertrophy with process elaboration, and functional alterations that shift astrocytic activities from homeostatic support toward proinflammatory responses (48, 49). Reciprocally, reactive astrocytes secrete a diverse array of inflammatory mediators, including additional cytokines, chemokines, and complement components, which further amplify microglial activation states and sustain inflammatory signaling, thereby establishing a self-perpetuating inflammatory feedback loop (50, 51). This bidirectional positive feedback signaling cascade substantially amplifies neuroinflammatory responses, creating a chronic inflammatory microenvironment within the CNS that may significantly contribute to the central neurological symptoms experienced by MG patients, particularly debilitating fatigue and cognitive dysfunction.

#### 3.2.2 Astrocyte phenotypic transformation

Activated microglia induce astrocyte conversion toward the neurotoxic A1 phenotype through secretion of TNF-α, IL-1α, and complement component C1q. A1 astrocytes are characterized by elevated expression of complement C3 and glial fibrillary acidic protein (GFAP), loss of normal functions including promotion of neuronal survival, synaptogenesis, and myelination, and acquisition of neurotoxic properties that enable them to kill neurons and oligodendrocytes (48–50). The neuroprotective A2 phenotype (expressing S100A10 and various neurotrophic factors) is significantly suppressed in chronic neuroinflammatory conditions (48, 50, 51). As shown in Table 1, microglia in MG predominantly polarize toward the M1 pro–inflammatory phenotype, whereas M2 anti–inflammatory markers are reduced. Similarly, astrocytes shift toward the neurotoxic A1 phenotype with downregulation of A2 markers.

**Table 1:** Glial cell phenotype characteristics and alterations in myasthenia gravis.

Phenotype	Markers	Primary functions	Alterations in MG	References
M1 microglia	CD86, iNOS, MHC-II, TNF-α, IL-1β	Pro-inflammatory response, neurotoxicity, antigen presentation	↑↑	(44–46)
M2 microglia	CD206, Arg-1, IL-10, TGF-β	Anti-inflammatory response, tissue repair, neuroprotection	↓	(46, 47)
A1 astrocytes	C3, GFAP↑, Serping1	Neurotoxicity, killing of neurons and oligodendrocytes	↑↑	(48–50)
A2 astrocytes	S100A10, PTX3, neurotrophic factors	Neuroprotection, promotion of synaptogenesis and myelination	↓	(48, 50, 51)

Arg-1, arginase-1; C3, complement component 3; GFAP, glial fibrillary acidic protein; iNOS, inducible nitric oxide synthase; MHC-II, major histocompatibility complex class II; MG, myasthenia gravis; PTX3, pentraxin 3; Serping1, serpin family G member 1; TGF-β, transforming growth factor-beta.

↑↑ indicates significantly increased; ↓ indicates decreased.

In summary, the aberrant activation and reciprocal interactions between microglia and astrocytes in MG collectively constitute a critical axis driving neuroinflammation and contributing to disease pathophysiology. The imbalance between M1/M2 and A1/A2 phenotypes results in the predominance of pro-inflammatory, neurotoxic phenotypes, while phenotypes with protective and reparative functions are suppressed. These glial cells collaborate to disrupt neuronal energy metabolism through impairment of glucose and lactate shuttling, perturb neurotransmitter homeostasis via alterations in glutamate clearance and synthesis, and directly generate neurotoxic substances including reactive oxygen and nitrogen species, ultimately culminating in progressive neuronal functional decline. The intricate network of glial cell interactions, characterized by overlapping signaling pathways and reciprocal activation patterns, represents a potential therapeutic vulnerability in MG. Targeting this glial cell interaction network through selective modulation of microglial activation states, enhancement of astrocytic neuroprotective functions, or interruption of pathological feedback loops may offer novel therapeutic strategies for intervening in CNS neuroinflammation associated with MG (52). Such approaches could potentially address the central neurological manifestations of MG that are inadequately controlled by conventional immunosuppressive therapies targeting peripheral immune responses.

### 3.3 Interactions between neuroinflammation and immune cell infiltration

In neurological disorders such as MG, neuroinflammation and immune cell infiltration establish a mutually reinforcing pathological cycle that amplifies tissue damage and perpetuates disease activity. This deleterious process is initiated by the activation of CNS-resident microglia, which release an array of cytokines and chemokines, including C-C motif chemokine ligand 2 (CCL2) and C-X-C motif chemokine ligand 10 (CXCL10), that not only amplify local inflammatory responses but, more critically, alter BBB permeability through disruption of tight junction integrity (53, 54). These inflammatory signals activate cerebrovascular endothelial cells, inducing upregulation of adhesion molecules such as vascular cell adhesion molecule-1 (VCAM-1), intercellular adhesion molecule-1 (ICAM-1), and E-selectin on the luminal endothelial surface. This molecular remodeling of the BBB facilitates the transmigration of peripheral immune cells, including T lymphocytes and monocytes/macrophages, across the vascular barrier and their subsequent infiltration into the CNS parenchyma (53, 54). Experimental and clinical evidence demonstrates that under neuroinflammatory conditions, the infiltration of peripheral immune cells substantially exacerbates local inflammation and tissue injury through sustained release of proinflammatory mediators, creating a positive feedback loop that perpetuates CNS pathology (53).

Infiltrating immune cells exert profound detrimental effects on the nervous system through both direct cytotoxic mechanisms and indirect inflammatory pathways that converge to impair neuronal function. Activated T cells and natural killer (NK) cells can directly induce neuronal injury through cytotoxic mechanisms, including perforin-granzyme-mediated apoptosis and Fas-FasL interactions that trigger programmed cell death pathways in target neurons. Concurrently, these infiltrating lymphocytes release interferon-gamma (IFN-γ) and other proinflammatory cytokines that further activate microglia and astrocytes, establishing complex tripartite cellular interactions that amplify inflammatory cascades and indirectly compromise neurological function through mechanisms including excitotoxicity, oxidative stress, and metabolic dysregulation (55, 56). This coordinated assault on neural tissue from multiple cellular sources creates a hostile microenvironment that impairs neuronal survival, synaptic function, and axonal integrity, ultimately manifesting as the neurological symptoms characteristic of MG.

Importantly, the relationship between neuroinflammation and infiltrating immune cells is not unidirectional; rather, neuroinflammatory signals exert reciprocal regulatory effects on immune cell function, dynamically modulating the phenotype and functional properties of infiltrating immune populations. Macrophages and T cells exhibit remarkable phenotypic plasticity within the neuroinflammatory milieu, with their polarization states critically determining the trajectory of neuroinflammatory progression. Under most pathological conditions, these cells polarize toward proinflammatory phenotypes, including classically activated M1-like macrophages and Th1/Th17-skewed T cells, which abundantly produce TNF-α, IL-6, IL-17, and other inflammatory mediators that perpetuate neuronal damage and sustain chronic inflammation (56, 57). This predominance of proinflammatory polarization creates a self-amplifying destructive cycle that drives progressive tissue injury. Conversely, under certain conditions, feedback regulatory mechanisms can redirect immune cell differentiation toward anti-inflammatory phenotypes, including alternatively activated M2-like macrophages and regulatory T cells (Tregs)/Th2-polarized cells. These immunoregulatory populations suppress inflammatory responses through secretion of anti-inflammatory cytokines such as interleukin-10 (IL-10) and transforming growth factor-beta (TGF-β), potentially limiting tissue damage and even promoting tissue repair and functional recovery (26, 58). The balance between these opposing polarization states represents a critical determinant of disease outcomes and a potential therapeutic target for modulating neuroinflammatory processes in MG.

Collectively, these findings illuminate the complex and dynamic nature of neuroinflammation in MG, characterized by intricate cellular interactions, bidirectional communication between the CNS and peripheral immune system, and the establishment of self-perpetuating inflammatory cascades. Understanding these mechanisms provides critical insights into potential therapeutic interventions that could target neuroinflammatory pathways to ameliorate both peripheral and central manifestations of MG, potentially improving patient outcomes beyond what is achievable with current immunosuppressive strategies alone.

## 4 Age-related neurodegenerative pathology and its mechanisms

### 4.1 Age-related changes in neuronal structure and function

**Figure 3:**
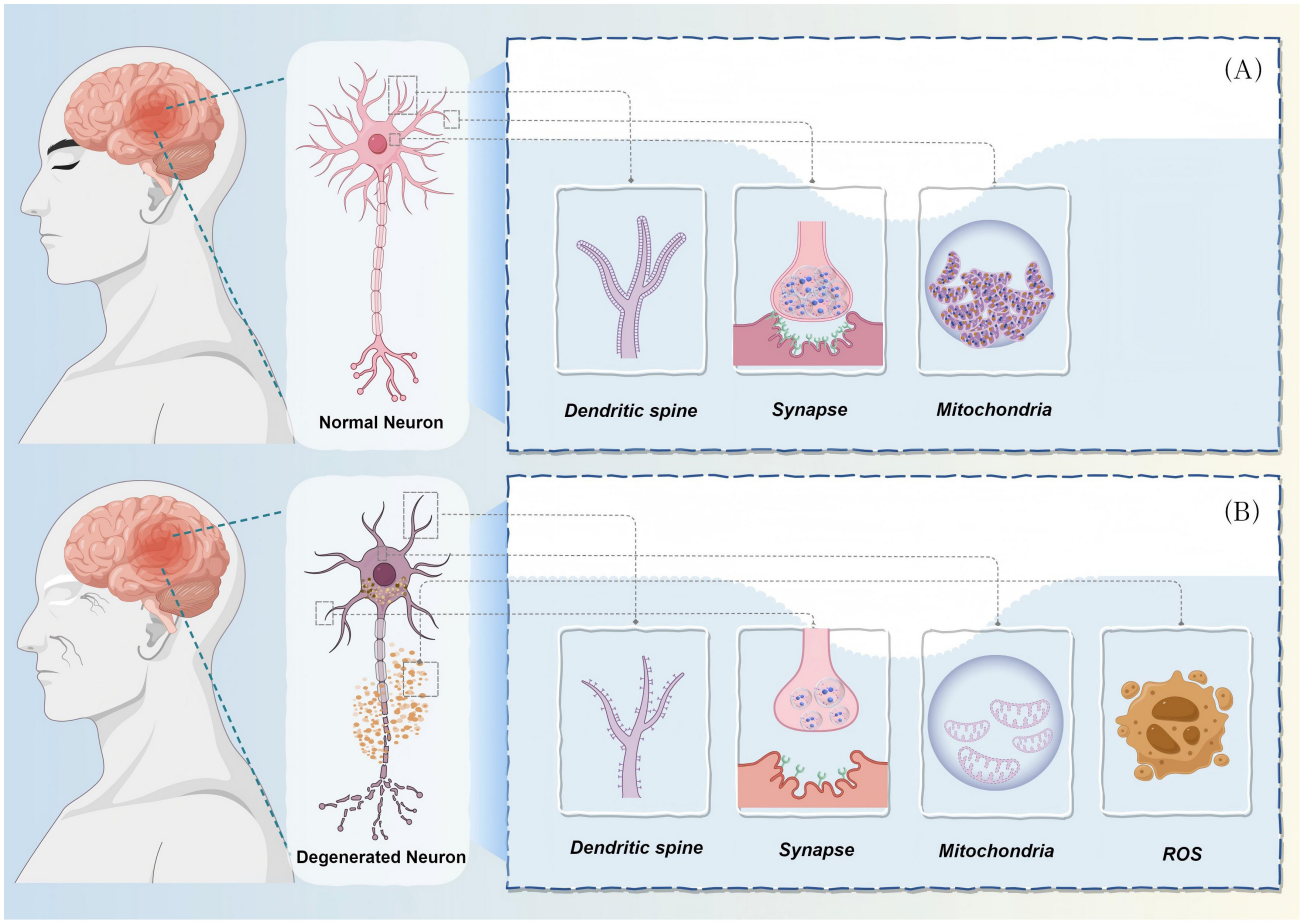
Ultrastructural and functional comparison between normal and degenerating neurons. **(A)** Structural and functional characteristics of normal neurons: healthy neurons exhibit complete neuronal morphology and optimal cellular functional states. Dendrites are densely populated with morphologically regular dendritic spines displaying typical mushroom-shaped, thin, or stubby configurations, with optimized surface areas to support efficient synaptic transmission. Synaptic structures are intact, including: abundant synaptic vesicles within presynaptic terminals, clearly defined synaptic clefts (approximately 20–40 nm), and well-organized postsynaptic density (PSD). Mitochondria display normal morphology with regular elliptical or rod-like structures containing well-ordered, densely packed cristae, indicating robust oxidative phosphorylation capacity. These mitochondria are evenly distributed throughout axons, dendrites, and soma, ensuring adequate ATP supply. The nucleus maintains regular morphology with uniform chromatin distribution and intact nuclear envelope. The cytoskeletal network (comprising microtubules, microfilaments, and intermediate filaments) is well-organized, maintaining normal cell morphology, intracellular transport, and signal transduction functions. Overall, normal neurons sustain efficient neurotransmitter release, synaptic plasticity, and energy metabolic homeostasis. **(B)** Pathological alterations and molecular mechanisms in degenerating neurons: under pathological conditions such as oxidative stress, neuroinflammation, or genetic mutations, neurons undergo progressive degenerative changes characterized by multiple interconnected pathological features: dendritic and synaptic pathology: dendritic spines exhibit significant numerical reduction (loss of 30%−70%) and morphological abnormalities, manifesting as spine neck elongation, head atrophy, or aberrant filopodial protrusions. Dendritic branching complexity decreases, with dendritic atrophy and pruning. Synaptic structural integrity is compromised, with presynaptic vesicle pool depletion, synaptic cleft widening, and postsynaptic density dissolution or disappearance, resulting in drastically reduced synaptic transmission efficiency and loss of synaptic plasticity. Mitochondrial dysfunction and oxidative stress cycle: mitochondria display severe morphological abnormalities, including excessive fragmentation (due to dysregulated fission-fusion dynamics), swelling, and cristae disorganization or loss. Mitochondrial membrane potential (ΔΨm) significantly decreases, electron transport chain function is impaired, leading to ATP insufficiency and energy crisis. More critically, dysfunctional mitochondria become a major source of reactive oxygen species (ROS), generating superoxide anions (${\text{O}}_{2}^{-}$) and other free radicals through electron leakage. This excessive ROS further damages mitochondrial DNA, lipids, and proteins, exacerbating mitochondrial dysfunction and establishing a self-reinforcing vicious cycle. Cascade effects of oxidative damage: accumulated ROS causes widespread oxidative injury, including: (i) lipid peroxidation, particularly in neuronal membranes rich in polyunsaturated fatty acids; (ii) protein carbonylation and aggregation, forming dysfunctional protein aggregates; (iii) DNA oxidative damage, including base modifications in both nuclear and mitochondrial DNA; (iv) depletion or inactivation of antioxidant defense systems (such as superoxide dismutase and glutathione systems). Organellar pathological changes**:** endoplasmic reticulum stress and unfolded protein response (UPR) activation, Golgi fragmentation, autophagy-lysosome system dysfunction leading to impaired clearance of damaged organelles and misfolded proteins. Cytoskeletal network disintegration, abnormal modification of microtubule-associated proteins, and axonal transport blockade. Ultimate consequences: the cumulative effects of these pathological changes lead to loss of neuronal synaptic function, disruption of neural network connectivity, and ultimately trigger neuronal apoptosis or necrotic cell death. This figure clearly illustrates the key pathological features in the transition from normal to degenerating neurons, revealing the core pathological loop of oxidative stress-mitochondrial dysfunction-neuronal injury, a mechanism with common significance across multiple neurodegenerative diseases including Alzheimer's disease, Parkinson's disease, and amyotrophic lateral sclerosis. Created with Figdraw (www.figdraw.com).

With advancing age, as shown in Figure 3, both the quantity and function of neurons undergo significant changes that profoundly impact the overall health and function of the nervous system. At the structural level, the aging brain does not simply exhibit widespread global neuronal loss; rather, it more commonly manifests as reduced dendritic spine complexity, aberrant axonal morphology, and extensive synaptic connectivity remodeling (59, 60). These subtle yet cumulative structural impairments directly compromise the stability and efficiency of neural networks, subsequently leading to declines in higher-order functions such as learning, memory, and motor coordination.

At the functional level, synaptic efficacy downregulation represents one of the most prominent features of aging. As critical nodes for information transmission, synapses experience significantly diminished plasticity with advancing age, which not only affects neural signal transmission efficiency but also directly constrains learning and memory formation (59, 60). Further research demonstrates that decreased synaptic density and reduced neurotransmitter release efficiency are closely associated with cognitive impairment during aging, manifesting as attenuated long-term potentiation (LTP) capacity and altered postsynaptic receptor expression (60, 61).

One crucial biological foundation underlying these degenerative changes is the progressive decline in axonal transport efficiency. Axonal transport is responsible for the long-distance delivery of mitochondria, neurotransmitter vesicles, neurotrophic factors, and other essential substances to presynaptic terminals. During aging, the efficacy of this transport system significantly decreases, particularly affecting brain-derived neurotrophic factor (BDNF)-dependent signaling endosome axonal transport (61), resulting in insufficient energy and material supply to synaptic sites, thereby directly impairing neural signal generation and transmission. This transport dysfunction is believed to be closely associated with multiple aging-related factors, including mitochondrial functional decline, intracellular calcium homeostasis disruption, dynein dysfunction, and exacerbated oxidative stress (60, 61), collectively forming a vicious cycle that leads to neuronal dysfunction and ultimately cell death.

### 4.2 Cumulative effects of cerebral inflammation and oxidative stress

Age-associated chronic low-grade inflammation, termed inflammaging, constitutes one of the core driving factors of neurodegenerative pathology. Within the central nervous system, this process manifests as sustained activation of microglia and astrocytes. While these cells play protective roles during acute injury, in chronic states they release excessive pro-inflammatory factors including tumor necrosis factor-α (TNF-α), interleukin-1β (IL-1β), and interleukin-6 (IL-6), directly damaging neurons and disrupting their function. This mechanism is particularly critical in cognitive decline associated with Alzheimer's disease (AD) and other conditions (62, 63). The cholinergic neurotransmitter system plays an important role in regulating aging-associated glial cell function, and its dysfunction further exacerbates neuroinflammation (62).

More importantly, chronic neuroinflammation and oxidative stress constitute a mutually driving dynamic process. On one hand, activated glial cells generate substantial reactive oxygen species (ROS) through pathways such as nicotinamide adenine dinucleotide phosphate (NADPH) oxidase, triggering oxidative stress. On the other hand, ROS themselves can act as signaling molecules to activate pro-inflammatory pathways such as nuclear factor-κB (NF-κB), further amplifying the inflammatory response (63). This mutual reinforcement between inflammation and oxidation significantly accelerates the deposition and toxicity of pathological proteins such as β-amyloid in diseases like AD, forming pathological cascade reactions.

The persistence of this state ultimately leads to neuronal damage and synaptic function loss through multiple mechanisms. Elevated ROS levels directly attack lipids, proteins, and DNA, causing widespread cellular structural damage, including lipid peroxidation, protein carbonylation, and oxidative DNA damage (63). In AD, the oxidative stress environment promotes oligomerization and accumulation of β-amyloid, thereby forming a neurotoxic microenvironment (62). Simultaneously, redox imbalance disrupts endogenous antioxidant defense pathways such as nuclear factor erythroid 2-related factor 2-antioxidant response element (Nrf2-ARE), weakening cellular self-protection capacity (64).

Ultimately, the deleterious microenvironment co-shaped by inflammation and oxidative stress poses severe threats to neuronal survival and function. Activated glial cells continuously release oxidants and pro-inflammatory factors, not only directly inducing neuronal death but also causing impaired synaptic plasticity and even synaptic loss, which constitutes the structural basis for cognitive and memory impairment (63, 65). The activation of microglial transforming growth factor-β-activated kinase 1 (TAK1) signaling pathway plays a critical role in promoting neurotoxic astrocyte phenotype transformation and cognitive dysfunction (65). Therefore, combined interventions targeting oxidative stress and inflammation, such as using antioxidants in conjunction with anti-inflammatory treatments, may provide new insights and hope for the prevention and treatment of neurodegenerative diseases.

### 4.3 Mitochondrial dysfunction and apoptosis

Mitochondria serve as the cellular powerhouse, and their dysfunction is closely associated with the pathological progression of various diseases, including myasthenia gravis (MG). In MG, research indicates that mitochondrial damage leads to energy metabolism disorders, subsequently affecting the normal function of neurons and muscle cells. Abnormal expression of mitophagy-related genes is widely observed in MG patients, and these genetic signatures are closely related to disease diagnosis and immune status (20). In MG patients, mitochondrial damage can result in insufficient adenosine triphosphate (ATP) synthesis, directly causing energy supply failure at the neuromuscular junction, thereby exacerbating muscle weakness and fatigue symptoms (21).

Beyond energy crisis, dysfunctional mitochondria become excessive sources of intracellular ROS, triggering severe oxidative stress. When mitochondrial respiratory chain function is impaired, electron transfer efficiency decreases, leading to excessive generation of superoxide anions and other ROS. Concurrently, compromised mitochondrial membrane potential (ΔΨm) stability also results in calcium ion homeostasis dysregulation (66). The combined effects of ROS accumulation and calcium overload can initiate the intrinsic apoptotic program via the mitochondrial pathway.

The hallmark events of this pathway include the opening of the mitochondrial permeability transition pore (mPTP) and cytochrome c release. Cytochrome c entering the cytoplasm binds with apoptotic protease activating factor-1 (Apaf-1) to form the apoptosome, activating the downstream caspase cascade, ultimately executed by caspase-3 to complete apoptosis (66). In muscle denervation models, deletion of the nucleotide-binding oligomerization domain-like receptor protein 3 (NLRP3) inflammasome can attenuate muscle atrophy by inhibiting pyroptosis, proteolysis, and apoptosis, suggesting the important role of the inflammasome-mitochondrial apoptosis axis in muscle degenerative changes (42). In MG, excessive activation of this process may lead to motor nerve terminal degeneration and muscle fiber loss, further compromising the structural and functional integrity of the neuromuscular junction (66, 67).

Therefore, mitochondrial protective strategies hold significant therapeutic promise. These include using antioxidants (such as coenzyme Q10 and vitamin E) to mitigate ROS damage, promoting mitophagy to clear damaged mitochondria, stabilizing mitochondrial membrane potential, or exploring caspase inhibitors to block apoptotic signal transduction. The development of nanodrug delivery systems provides new possibilities for precision mitochondrial-targeted therapy (68), offering potential as an emerging therapeutic direction for delaying MG disease progression and protecting neuromuscular function.

### 4.4 Interactions between neuroimmune inflammation and age-related degenerative pathology

#### 4.4.1 Impact of immune system aging on MG pathogenesis

Immunosenescence refers to the functional decline of the immune system with advancing age, with immune regulatory network imbalance as one of its core features. Epidemiological studies have confirmed that advanced age is an important risk factor for MG, with the proportion of late-onset MG (onset ≥50 years) among all MG patients showing a rising trend (5, 23).

**Figure 4:**
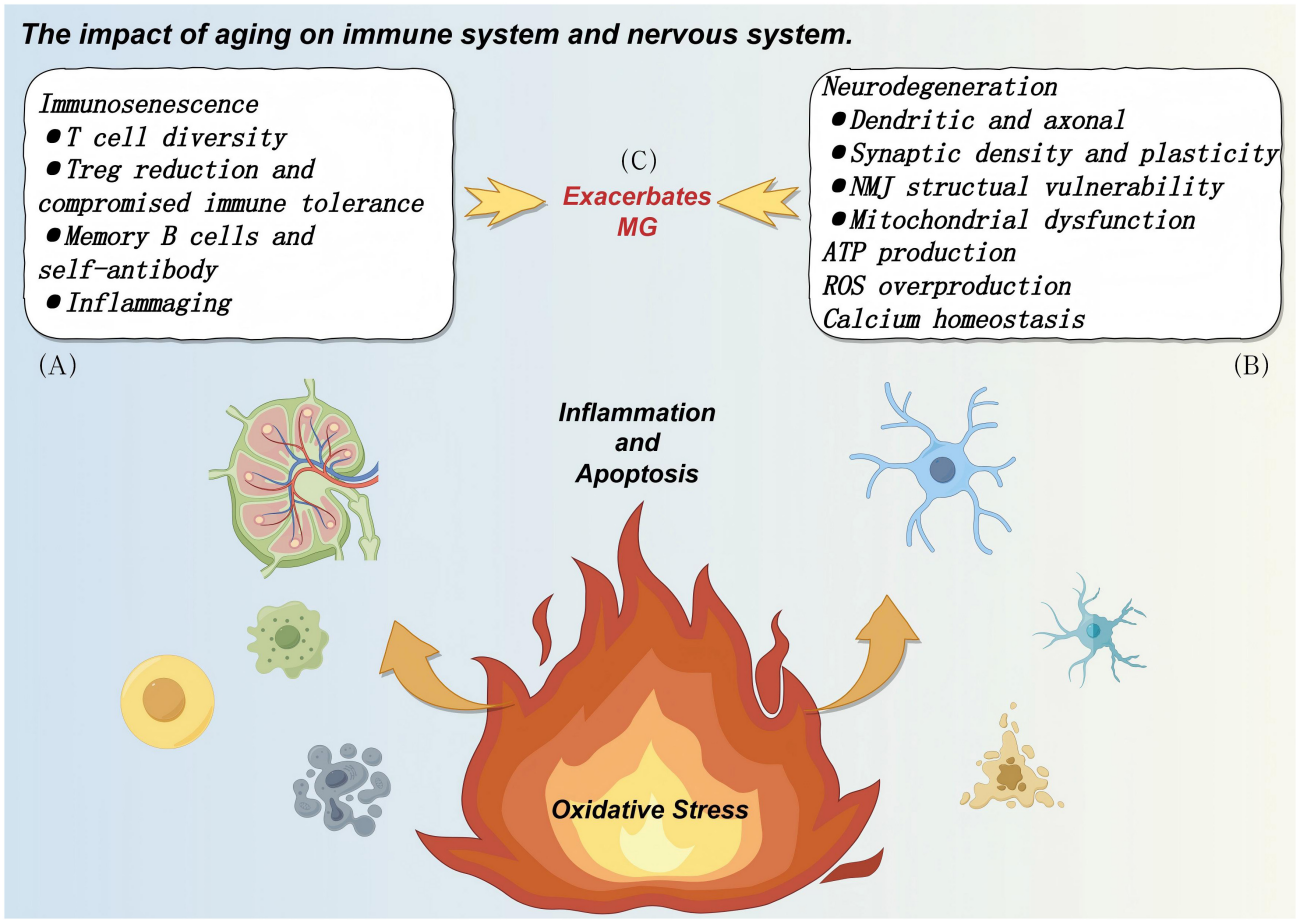
Aging-driven dual-track degeneration of immune and nervous systems and their synergistic mechanisms in myasthenia gravis pathogenesis. Aging drives myasthenia gravis (MG) pathogenesis through two interconnected pathways—immunosenescence and neurodegeneration—that form a self-reinforcing cycle culminating in neuromuscular junction (NMJ) structural and functional failure. **(A)** Immunosenescence pathological alterations: aging induces comprehensive immune system dysfunction through multiple mechanisms: thymic involution and T cell dysfunction: progressive thymic atrophy (reducing to 10%−15% of original volume by age 60) drastically decreases naive T cell generation and T cell receptor (TCR) repertoire diversity, impairing immune recognition capacity and skewing toward pro-inflammatory phenotypes. Immune tolerance collapse: despite maintained numbers, regulatory T cells (Tregs) exhibit impaired suppressive function with unstable Foxp3 expression and reduced IL-10/TGF-β secretion, causing both central and peripheral tolerance failure and allowing autoreactive T cell escape. B cell reprogramming and autoantibody production: altered B cell pool composition with abnormal germinal center reactions leads to selective expansion of autoreactive clones, directly causing pathogenic autoantibody production (anti-AChR, anti-MuSK, anti-LRP4) in MG. Inflammaging: senescence-associated secretory phenotype (SASP) factors create chronic low-grade systemic inflammation, maintaining autoimmune responses while increasing blood-nerve barrier permeability. Innate immune cells (macrophages, dendritic cells, NK cells) shift toward pro-inflammatory phenotypes with reduced regulatory functions. **(B)** Neurodegeneration and NMJ integrity loss: age-related nervous system changes cause comprehensive NMJ degeneration: neuronal morphological decline: motor neurons undergo reduced dendritic complexity (30–50% spine loss), axonal thinning, segmental demyelination, and 30–40% neuronal loss by age 70, directly impairing signal transmission to NMJ. Synaptic dysfunction**:** decreased synaptic vesicle numbers and cycling efficiency, reduced active zones and calcium channel function, lowered postsynaptic AChR density, and impaired synaptic plasticity (LTP/LTD) collectively reduce NMJ transmission efficiency and adaptability. NMJ structural fragility: postsynaptic fold simplification, reduced contact area, widened synaptic cleft, synaptic uncoupling, endplate fragmentation, and declined Schwann cell function render NMJ highly vulnerable to immune attack. Mitochondrial dysfunction cascade**:** aging mitochondria exhibit decreased oxidative phosphorylation efficiency causing ATP insufficiency, increased electron leakage producing excessive ROS, impaired calcium buffering causing homeostasis disruption, and failed quality control (mitophagy) leading to damaged mitochondria accumulation. These alterations trigger oxidative stress-inflammation-apoptosis vicious cycles, causing lipid peroxidation, protein aggregation, DNA damage, NF-κB activation, and ultimately motor neuron apoptosis, resulting in postsynaptic membrane destruction and AChR loss. **(C)** Synergistic amplification effects: immunosenescence and neurodegeneration interact through multiple mechanisms forming a “double hit”: targeted immune attack**:** autoreactive lymphocytes specifically target already-fragile NMJ self-antigens, with tolerance defects preventing clearance. Pathogenic autoantibodies destroy vulnerable NMJ through CDC, ADCC, and receptor internalization mechanisms. Inflammatory toxicity amplification**:** systemic pro-inflammatory cytokines (TNF-α, IL-1β, IL-6) penetrate compromised blood-nerve barriers, producing additional toxicity on energy-deficient, oxidatively-stressed aging neurons, while activating glia to establish persistent neuroinflammatory microenvironments. Metabolic-immune vicious cycle: aging neurons release DAMPs (mtDNA, ATP, HMGB1) that activate innate immunity via TLRs and inflammasomes, creating positive feedback loops amplifying NMJ destruction. Dual repair failure: combined immunosenescence (reduced M2 macrophages, Tregs) and neural aging (decreased axonal regeneration, synaptic plasticity, satellite cell potential) mean even mild immune attacks cause irreversible damage. This dual-track degeneration model explains MG's elderly onset peak (60–80 years) with severe symptoms and poor treatment responses, while providing theoretical foundations for precision therapies combining immunomodulation and neuroprotection in elderly MG patients. Created with Figdraw (www.figdraw.com).

Figure 4 illustrated aging-driven dual-track degeneration of immune and nervous systems. Due to the older age of late-onset MG patients, the intrinsic immune characteristics of MG often exhibit nested relationships with the immunosenescence phenotype. At the cellular immunity level of immunosenescence, manifestations include reduced T cell repertoire diversity and functional skewing toward pro-inflammatory phenotypes. Specifically, this presents as increased proportions of effector memory T cells, while regulatory T cells (Tregs) with immunosuppressive functions exhibit compromised quantity and function (69, 70). At the humoral immunity level, B cell function undergoes remodeling, with abnormal activation of autoreactive subsets such as age-associated B cells (ABCs), which is closely related to the titer of acetylcholine receptor antibody (AChR-Ab) (71). Furthermore, immunosenescence is commonly accompanied by a systemic pro-inflammatory state, i.e., “inflammaging,” characterized by persistently elevated pro-inflammatory cytokines (such as IL-6, TNF-α, IL-1β), with relatively insufficient anti-inflammatory factors (such as interleukin-10 [IL-10] and transforming growth factor-β [TGF-β]) (72, 73). Alterations in post-translational modifications play important roles during immunosenescence, affecting antigen presentation and autoantibody production (72, 73). This microenvironment further disrupts immune tolerance and drives autoimmune responses.

Sex differences also play important roles in immunosenescence and MG pathogenesis. Immunometabolic reprogramming exhibits significant sex differences, with females showing higher MG incidence at younger ages, while sex differences in elderly MG diminish, potentially related to immune regulatory changes caused by declining estrogen levels (69, 70).

In summary, immunosenescence leads to immune homeostasis disruption through effects on T cell and B cell function, cytokine networks, and immune metabolism, significantly increasing MG susceptibility and potentially resulting in more aggressive clinical presentations and differential treatment responses in elderly patients (74). In-depth understanding of these specific immune features will provide important theoretical foundations for personalized immunotherapy strategies in elderly MG patients. Particularly important is that this aging-associated immune imbalance provides conditions for the initiation and maintenance of neuroinflammation and produces complex interactions with age-related neurodegeneration.

##### 4.4.1.1 Evidence linking peripheral immunosenescence to central pathology

Accumulating evidence suggests an association between peripheral immunosenescence features and central nervous system (CNS) pathological status in patients with LOMG. At the cellular immunity level, the proportion of CD4^+^CD28^−^ T cells is significantly elevated in the peripheral blood of LOMG patients. These cells exhibit pro-inflammatory properties and have been implicated in the pathogenesis of various autoimmune diseases (75, 76). Studies have demonstrated that the expansion of CD4^+^CD28^−^ T cells is negatively correlated with cognitive function scores (*r* = −0.42, *P* < 0.05) (76, 77). At the inflammatory mediator level, elevated serum IL-6 levels represent a prominent feature in LOMG patients and are positively correlated with fatigue severity (*r* = 0.56, *P* < 0.01) (3). IL-6 can cross the blood-brain barrier or act on cerebrovascular endothelial cells through multiple pathways, thereby promoting central neuroinflammation (78). Furthermore, reduced regulatory T cell (Treg) function is negatively correlated with disease severity (*r* = −0.38, *P* < 0.05) (79). At the cellular senescence biomarker level, telomere length, a classic biomarker of cellular senescence, exhibits a shortening trend in LOMG patients and is associated with late age at onset (80, 81). Limitations and future directions: it should be noted that studies directly examining cerebrospinal fluid (CSF) inflammatory markers or CNS imaging alterations in LOMG patients remain limited, and the aforementioned peripheral-central associations are largely based on indirect inference. Future clinical studies incorporating CSF biomarker analysis, functional neuroimaging assessment, and longitudinal follow-up designs are urgently needed to establish direct associations between peripheral immunosenescence and central pathology (82). Table 2 provides an overview of the associations between peripheral immunosenescence markers and central/clinical indicators in LOMG patients.

**Table 2:** Associations between peripheral immunosenescence markers and central/clinical indicators in LOMG patients.

Peripheral marker	Central/ Clinical indicator	Correlation	References
CD4^+^CD28^−^ T cells ↑	Cognitive function scores ↓	*r* = −0.42^*^	(76, 77)
Serum IL-6 ↑	Fatigue severity ↑	*r* = 0.56^**^	(83, 84)
Treg function ↓	Disease severity ↑	*r* = −0.38^*^	(79)
Telomere length ↓	Late age at onset	Associated	(80, 81)

IL-6, interleukin-6; LOMG, late-onset myasthenia gravis; Treg, regulatory T cell.

^*^P < 0.05; ^**^P < 0.01.

↑ indicates increased; ↓ indicates decreased.

#### 4.4.2 Mechanisms by which degenerative pathology exacerbates neuroinflammatory responses

Neurodegenerative pathology and neuroinflammatory responses often constitute self-reinforcing vicious cycles. During pathological processes, damaged or dying neurons release various damage-associated molecular patterns (DAMPs), including pathological proteins (such as β-amyloid, α-synuclein, tau protein), extracellular ATP, high mobility group box 1 (HMGB1), and other signaling molecules (83, 84). These substances can act as endogenous ligands to activate pattern recognition receptors [PRRs; such as Toll-like receptors and receptor for advanced glycation end products (RAGE)] on microglial and astrocyte surfaces, triggering neuroinflammation and releasing pro-inflammatory factors including TNF-α, IL-1β, and IL-6 (22, 83). These factors not only exacerbate local inflammation but also can directly cause further damage and dysfunction of adjacent neurons through multiple mechanisms, including inducing oxidative stress, disrupting calcium homeostasis, impairing mitochondrial function, and interfering with synaptic plasticity.

More importantly, this process forms a sustained feedback loop. The release of inflammatory mediators promotes additional cellular damage and apoptosis; while newly apoptotic neurons release more DAMPs, further activating glial cells and triggering more intense inflammatory responses, ultimately resulting in continuous neuronal loss (22, 83). This “inflammation-damage” cycle constitutes the common pathological basis of multiple neurodegenerative diseases (such as Alzheimer's disease, Parkinson's disease, and amyotrophic lateral sclerosis) (22, 84). The chemokine system plays a critical role in this cycle; for example, blockade of the CC chemokine ligand 21-CC chemokine receptor 7 (CCL21-CCR7) signaling axis can prevent neuroinflammation and neurodegeneration in Parkinson's disease models (22, 83).

Thus, complex bidirectional regulatory relationships exist between immune cell activation and neurodegenerative changes. On one hand, degenerative pathology activates both central and peripheral immune cells, including microglia, astrocytes, as well as infiltrating peripheral monocytes and T cells. On the other hand, the activation state of immune cells profoundly influences neuronal fate: moderate activation helps clear cellular debris, phagocytose pathological proteins, and provide neurotrophic support [such as secreting BDNF and insulin-like growth factor-1 (IGF-1)]; however, once dysregulated, excessive inflammation dominates the process, accelerating neuronal death and disease progression through the release of pro-inflammatory factors, ROS, and proteases (83, 84). Activation of triggering receptor expressed on myeloid cells 1 (TREM1) in myeloid immune cells disrupts myeloid cell bioenergetics and exacerbates cognitive dysfunction (22, 84). This duality suggests that future therapeutic strategies must focus on precisely modulating immune responses, aiming to suppress harmful inflammation while preserving its protective functions, thereby opening new avenues for effectively improving neurodegenerative disease prognosis (83, 84).

#### 4.4.3 Pathological processes by which neuroinflammation accelerates degenerative pathology

In MG, persistent autoimmune responses may lead to excessive release of inflammatory mediators [such as TNF-α, IL-1β, IL-6, and interleukin-17 (IL-17)]. These factors not only exert effects peripherally but may also influence the central nervous system through multiple pathways. On one hand, they can directly enter brain parenchyma through regions with incomplete blood-brain barriers such as circumventricular organs; on the other hand, peripheral inflammatory signals can be transmitted to the central nervous system through neuroimmune pathways such as the vagus nerve (6). These pathways collectively activate endogenous immune cells (such as microglia and astrocytes), initiating and maintaining neuroinflammatory responses (44, 84). Hypoxia-inducible factor-1α (HIF-1α) plays a critical role in this process, interacting with T helper 17 (Th17) cell activation to jointly promote neuroinflammation progression (84).

This inflammatory state mutually exacerbates oxidative stress, jointly disrupting neuronal homeostasis. Activated microglia produce substantial ROS through NADPH oxidase, while inflammatory factors also impair neuronal mitochondrial function, further increasing ROS production. The synergistic effects of oxidative stress and inflammation can activate multiple apoptotic signaling pathways, including both caspase-dependent and -independent pathways, directly leading to neuronal damage and death (21). Research demonstrates that extracellular vesicles loaded with caspase-1 inhibitors can ameliorate experimental autoimmune myasthenia gravis by targeting macrophages (44). Therefore, understanding the roles of these inflammatory mediators in neuronal damage and apoptosis is critical for developing novel therapeutic strategies.

This mechanism shares commonalities with classical neurodegenerative diseases. For instance, in Alzheimer's disease and Parkinson's disease, neuroinflammation serves as a core driving factor of pathological progression, with sustained microglial activation forming vicious cycles that exacerbate β-amyloid deposition, neurofibrillary tangles, and α-synuclein aggregation (85, 86). Fyn kinase plays important roles in the pathological processes of multiple neurodegenerative diseases and may serve as a universal therapeutic target (18, 87). Indeed, similar chronic neuroinflammation very likely exists in MG, which may also potentially accelerate functional neurodegeneration, partially explaining the central symptoms commonly observed in MG patients, such as fatigue and cognitive dysfunction.

Moreover, neuroinflammation damages nervous system homeostasis through multiple aspects by altering the neuronal microenvironment. First, inflammatory mediators can induce pyroptosis by activating gasdermin D (GSDMD) protein on endothelial cells, disrupting blood-brain barrier integrity and increasing neuronal exposure to peripheral harmful substances (41). Second, dysfunctional astrocytes under inflammatory conditions exhibit reduced glutamate clearance capacity and potassium ion buffering deficits, thereby increasing the risk of excitotoxic neuronal damage. Third, chronic inflammation disrupts normal communication among neurons, astrocytes, and vascular endothelial cells, compromising functional coordination of the neurovascular unit (88). This microenvironmental deterioration, through enhanced cumulative effects of oxidative stress and inflammatory damage, collectively forms a dynamic process driving neuronal apoptosis and degeneration.

The role of neuroinflammation in nervous system diseases is increasingly prominent, with its potentially mediated secondary neurodegenerative pathology in myasthenia gravis emerging as a new research focus (44). Understanding this process, particularly how peripheral autoimmunity influences central nervous system function through neuroinflammatory pathways, is crucial for comprehensively recognizing long-term neurological damage in MG and developing targeted neuroprotective therapeutic strategies.

## 5 Age-dependent pathophysiological mechanisms and neurodegenerative features of myasthenia gravis

Myasthenia gravis (MG), an autoimmune disorder of the neuromuscular junction, exhibits intricate and complex interactions between age-related immunosenescence and neurodegenerative changes in its pathophysiology (1, 89). The clinical phenotypic heterogeneity observed across different age groups in MG patients fundamentally reflects the dynamic interplay among immune system aging, thymic involution, neuromuscular junction structural degeneration, and the evolution of autoantibody profiles (1, 90). Understanding these age-dependent pathophysiological alterations is crucial for elucidating the pathogenesis of MG, optimizing diagnostic and therapeutic strategies, and predicting disease progression.

### 5.1 Age-related immunosenescence and MG pathogenesis

#### 5.1.1 Adolescence: active thymic function and autoimmune susceptibility

Pediatric and adolescent MG ( ≤ 18 years) accounts for approximately 10%−15% of all cases, with distinctive clinical features closely related to the active thymic hematopoietic function and developing immune system characteristic of this age group (91). Prepubertal children demonstrate a marked predilection for ocular MG (60%−70%), predominantly affecting extraocular muscles with manifestations of ptosis and diplopia, while rarely progressing to generalized disease (92, 93). This ocular-predominant pattern likely reflects developmental specificity of the neuromuscular junction, unique metabolic characteristics of extraocular muscle fibers, and age-specific T cell repertoire and B cell response patterns (94).

From an immunological perspective, the serological profile of pediatric MG reveals a lower acetylcholine receptor (AChR) antibody positivity rate (50%−60%) compared to adults, with a substantial proportion of patients being seronegative, suggesting the potential existence of unidentified autoantigens or non-antibody-mediated immune mechanisms (95, 96). Thymic histological examination predominantly shows follicular hyperplasia, characterized by abundant germinal centers in the medullary region, B cell infiltration, and plasma cell accumulation, constituting a critical site for anti-AChR antibody synthesis (30, 97). Notably, approximately 30%−40% of prepubertal patients can achieve complete remission without immunosuppressive therapy, a high spontaneous remission rate that may reflect the plasticity of the developing immune system and its capacity for immune tolerance reconstitution, which is exceedingly rare in adult MG (92, 93).

Transient neonatal MG represents a unique clinical phenomenon caused by transplacental transfer of maternal IgG antibodies, affecting 10%−20% of infants born to AChR antibody-positive mothers (98). Neonates present with generalized weakness, feeding difficulties, respiratory insufficiency, and weak cry, with symptoms typically resolving spontaneously within 2–3 months as maternal antibodies undergo catabolism (97). This phenomenon vividly demonstrates the pathogenic nature of MG autoantibodies and their capacity for passive transfer.

#### 5.1.2 Early adult onset: sex hormone modulation and immune homeostasis disruption

Early-onset MG (EOMG; onset at 19–50 years) represents the first epidemiological peak of the disease, exhibiting significant female predominance (female:male ratio approximately 3:1), suggesting involvement of sex hormones and X-chromosome-linked immune genes in pathogenesis (98, 99). Estrogen modulates immune responses through multiple mechanisms, including influencing lymphocyte homing, regulating cytokine production, and modulating thymic function, potentially enhancing B cell survival, promoting antibody production, and increasing autoimmune susceptibility (97, 100). This hormone-mediated immune activity may explain the gender disparity in EOMG and disease activity fluctuations during pregnancy (approximately 30%−40% of pregnant women experience exacerbation, primarily during the first trimester and postpartum period) (98).

EOMG patients typically present with generalized weakness affecting ocular, bulbar, limb, and respiratory muscles, with approximately 85%−90% of patients initially presenting with ocular symptoms ultimately progressing to generalized disease. This generalization process typically occurs within the first 2 years following onset, establishing a critical therapeutic intervention window (1, 101). Serologically, EOMG demonstrates high AChR antibody positivity rates (85%−90%) correlated with disease severity (1, 90). Human leukocyte antigen (HLA) association studies reveal strong correlations with HLA-B8, DR3, and DQ2 haplotypes, particularly in populations of European ancestry (1, 90).

Approximately 80%−85% of EOMG patients exhibit thymic abnormalities, predominantly follicular thymic hyperplasia (70%−75%), with 10%−15% presenting with thymoma (30, 102). Hyperplastic thymus demonstrates active germinal center formation, indicating sustained antigen-driven immune responses within the thymic microenvironment, supporting the central role of the thymus in EOMG pathogenesis (30, 97).

#### 5.1.3 Late onset: convergence of immunosenescence and neurodegenerative changes

Late-onset MG (LOMG; onset ≥50 years) currently accounts for over 50% of newly diagnosed cases in developed countries, reflecting population aging and improved diagnostic awareness (74, 103). LOMG exhibits male predominance (male:female ratio approximately 2–3:1), reversing the gender distribution observed in EOMG and suggesting distinct pathophysiological foundations (99, 104).

The distinctive clinical phenotype of LOMG—characterized by rapidly progressive generalized weakness, more severe bulbar and respiratory muscle involvement, higher incidence of myasthenic crisis (25%−35%), and mortality rates (8%−15%)—fundamentally reflects the cumulative effects of multiple age-related factors (104, 105):

(1) Immunosenescence: with advancing age, T cell repertoire diversity decreases, regulatory T cell (Treg) function weakens, and chronic inflammatory states (inflammaging) intensify, leading to increased autoimmune susceptibility and immune regulatory imbalance (73, 106).
(2) Thymic involution: 80%−85% of LOMG patients exhibit physiological thymic fatty replacement (thymic involution) with loss of thymic epithelial space and reduced naive T cell production, though 15%−20% still harbor thymoma (predominantly B2 and B3 subtypes), which demonstrates higher malignant potential compared to younger patients (102, 107).
(3) Neuromuscular junction degenerative changes: with aging, presynaptic motor nerve terminal acetylcholine release decreases, postsynaptic membrane AChR density declines, and the safety factor of neuromuscular transmission diminishes, rendering the elderly population more vulnerable to autoantibody-mediated attack (17, 108).
(4) Autoantibody profile alterations: LOMG patients demonstrate significantly increased frequency (40%−50%) of anti-striated muscle antibodies—particularly anti-Titin and anti-Ryanodine receptor (RyR) antibodies—compared to < 5% in EOMG (109, 110). These striated muscle antibodies correlate with more severe generalized disease, greater bulbar involvement, and higher thymoma prevalence, potentially reflecting age-related muscle protein degradation and increased antigen exposure (109, 110).
(5) Comorbidity burden: age-related conditions including cardiovascular disease, diabetes, and chronic obstructive pulmonary disease reduce physiological reserve, limiting tolerance to respiratory muscle weakness and treatment adverse effects (111).

### 5.2 Age-dependent degenerative changes of the neuromuscular junction

#### 5.2.1 Synaptic structure and functional aging

Age-related degenerative changes of the neuromuscular junction provide an important structural basis for the age-dependent clinical features of MG (17, 108). During normal aging, the neuromuscular junction undergoes multilevel degenerative alterations: mitochondrial dysfunction in presynaptic motor nerve terminals leads to decreased ATP production, affecting acetylcholine synthesis and vesicular release (17, 108); postsynaptic membrane AChR density declines with age, with impaired expression and function of receptor clustering proteins (such as rapsyn and MuSK) (112); synaptic cleft basal lamina thickens with altered acetylcholinesterase (AChE) activity (108). These structural and functional changes collectively result in decreased neuromuscular transmission efficiency and reduced safety factor.

In MG patients, autoantibody-mediated pathological damage superimposes upon these age-related degenerative foundations (113). Elderly patients, having already reduced baseline neuromuscular transmission reserves, are more vulnerable to equivalent levels of autoantibody attack, which may explain why LOMG patients often exhibit more severe clinical symptoms despite similar antibody titers compared to younger patients (89, 105). Single-fiber electromyography (SFEMG) studies demonstrate that healthy elderly individuals exhibit slightly higher jitter values compared to younger controls, reflecting age-related increased neuromuscular transmission instability, necessitating age-adjusted normal values when interpreting SFEMG results in elderly MG patients (114).

#### 5.2.2 Age-related differences in muscle fiber types and metabolic characteristics

The selective vulnerability of different muscle groups in MG is partly related to their fiber type composition and metabolic characteristics, which vary with age (89). Extraocular muscles contain unique fast fatigable muscle fibers with high neuromuscular junction density but relatively limited AChR reserves, rendering them particularly sensitive to antibody-mediated receptor blockade or destruction (94). Elderly patients' muscles undergo sarcopenia-related changes, including preferential atrophy of fast muscle fibers (type II), conversion of type II to type I fibers, mitochondrial dysfunction, and increased oxidative stress, which may influence the muscle involvement pattern and disease severity in MG (115).

### 5.3 Age-stratified diagnostic strategies

#### 5.3.1 Age-specific interpretation of serological testing

Serological diagnosis of MG requires consideration of age-related antibody profile differences (90, 116). Pediatric patients exhibit higher seronegativity rates (40%−50%), necessitating comprehensive antibody testing including AChR antibodies (binding, modulating, and blocking types), muscle-specific kinase (MuSK) antibodies, and low-density lipoprotein receptor-related protein 4 (LRP4) antibodies (95, 96). Binding antibody detection (radioimmunoprecipitation assay) demonstrates approximately 85% sensitivity in generalized MG but only 50% in pure ocular MG; modulating antibody testing may detect cases negative for binding antibodies, with 70%−80% sensitivity (94, 95).

EOMG patients exhibit peak AChR antibody positivity rates (85%−90%), while LOMG patients, despite similar AChR antibody positivity rates, demonstrate significantly elevated frequency of anti-striated muscle antibodies (Titin, RyR) at 40%−50% (109, 117). The presence of anti-striated muscle antibodies closely correlates with thymoma and more severe disease; detection of such antibodies in young patients should raise high suspicion for thymoma, even if imaging remains unremarkable (110). These antibodies may appear months to years before radiological detection of thymoma, suggesting the need for close follow-up monitoring (102, 110).

#### 5.3.2 Age-adjusted neurophysiological assessment

Repetitive nerve stimulation (RNS) demonstrates characteristic decremental responses (≥10%) in generalized MG with approximately 60%−80% sensitivity, but only 40%−50% in pure ocular MG (114). Testing multiple muscles (particularly facial, trapezius, and abductor digiti minimi) can enhance detection rates (118). Post-exercise facilitation and exhaustion phenomena provide additional diagnostic clues.

SFEMG represents the most sensitive electrodiagnostic technique, with abnormality rates of 95%−99% in generalized MG and 85%−95% in pure ocular MG (114). However, SFEMG abnormalities are not MG-specific, also occurring in other neuromuscular junction disorders (such as Lambert-Eaton myasthenic syndrome), neuropathies, and myopathies (114). As healthy elderly individuals exhibit slightly elevated jitter values compared to younger individuals, age-adjusted normal values should be employed when interpreting SFEMG results in elderly patients (114).

#### 5.3.3 Age-specific thymic imaging manifestations

Anterior mediastinal imaging assessment is crucial for identifying thymoma, evaluating thymic hyperplasia, and guiding therapeutic decisions (119). Computed tomography (CT) and magnetic resonance imaging (MRI) each offer advantages, with CT providing excellent spatial resolution and MRI superior soft tissue contrast without radiation exposure, making it more suitable for serial monitoring in children and young adults (119). Children and adolescents typically display normal thymus or diffuse enlargement (follicular hyperplasia), with thymoma being exceedingly rare (< 5%) (30). EOMG patients demonstrate heterogeneous imaging findings, ranging from normal to marked hyperplasia or discrete thymoma (10%−15%) (30, 102). LOMG patients show fatty involution (physiological atrophy) in 80%−85%, though 15%−20% harbor thymoma, often more aggressive B2 and B3 subtypes (102, 120).

### 5.4 Age-specific treatment response and prognostic mechanisms

#### 5.4.1 immunological basis of thymectomy efficacy

Age-related differences in thymectomy efficacy reflect functional variations in thymic immune function across age groups (121, 122). Children and adolescents (particularly those with thymic hyperplasia and generalized disease) derive significant benefit from thymectomy, achieving complete stable remission rates of 40%−50% and marked improvement rates of 35%−40%, with total benefit rates exceeding 80%−85% (122, 123). These excellent outcomes stem from active thymic hematopoiesis and antibody production within the hyperplastic thymus in young patients, with thymectomy effectively eliminating the primary source of pathogenic antibody synthesis and autoreactive lymphocytes (97).

EOMG patients (18–50 years) demonstrate thymectomy efficacy confirmed by the MGTX (Thymectomy Trial in Non-thymomatous Myasthenia Gravis Patients Receiving Prednisone Therapy) randomized controlled trial, showing that extended thymectomy combined with standard immunosuppression, compared to immunosuppression alone, reduces MG scores, decreases prednisone requirements (65% reduction in 3-year cumulative dose), and increases rates of achieving minimal manifestation status (124). Approximately 30%−40% of patients require only cholinesterase inhibitor maintenance at 3 years post-surgery (34).

Conversely, LOMG patients (≥50 years) derive limited benefit from thymectomy, with complete stable remission rates of only 15%−25% and 30%−40% achieving partial improvement while still requiring immunosuppression (121). This reflects markedly diminished immune function of the involuted fatty thymus, predominance of peripheral autoimmune mechanisms, and increased surgical risks with age and comorbidities (111). Current consensus recommends considering surgery for LOMG patients with early disease course (< 5 years), few comorbidities, and imaging evidence of thymic abnormalities, but exercising caution in those >70 years or with significant cardiopulmonary disease (101, 121).

#### 5.4.2 Age-related considerations in immunosuppressive therapy

Age-specific pharmacotherapy considerations primarily involve pharmacokinetic differences and adverse effect susceptibility (101). Cholinesterase inhibitors (pyridostigmine) serve as first-line symptomatic treatment across all age groups, though elderly patients demonstrate increased sensitivity to cholinergic side effects (abdominal pain, diarrhea, and increased salivation), often requiring dose reduction or slow titration; children may require relatively higher weight-adjusted doses due to faster metabolism (89, 101).

Corticosteroids constitute the cornerstone of MG immunosuppression, but adverse effects demonstrate significant age-related variation, determining treatment strategies (101, 111). Children face risks of growth retardation, delayed sexual development, and decreased bone density with long-term use, necessitating early introduction of steroid-sparing immunosuppressants (azathioprine, mycophenolate mofetil) (92, 125). Elderly patients experience dramatically increased corticosteroid-related risks, including osteoporosis, fractures, hyperglycemia/diabetes, hypertension, cardiovascular events, infections, and cognitive impairment (101, 111), warranting lower initial doses (0.5 mg/kg/day) and more gradual escalation (101).

Steroid-sparing immunosuppressants (azathioprine, mycophenolate mofetil, cyclosporine, etc.) play critical roles in minimizing steroid exposure while maintaining disease control (92, 125). Azathioprine demonstrates efficacy in 60%−80% of patients but requires 6–12 months for onset (1, 90); mycophenolate mofetil exhibits faster onset (3–6 months) (125). Elderly patients using immunosuppressants require particular attention to infection risks, dose adjustments based on renal and hepatic function, and vigilance for drug interactions (101).

Novel targeted therapies—including rituximab (anti-CD20 monoclonal antibody), complement inhibitors (such as eculizumab and ravulizumab), and neonatal Fc receptor (FcRn) inhibitors (such as efgartigimod and rozanolixizumab)—provide new options for refractory MG, though age-specific efficacy and safety data continue to accumulate (126–129).

#### 5.4.3 Age-stratified prognostic features

Long-term prognosis in MG demonstrates significant age gradients (98, 99). Adolescents exhibit the most favorable prognosis, with 40%−50% achieving complete stable remission and an additional 30%−40% maintaining excellent functional status (91). Favorable prognostic predictors include prepubertal onset, pure ocular MG, thymic hyperplasia without thymoma, low antibody titers, and early thymectomy (97).

EOMG patients demonstrate intermediate prognosis, with 30%−40% achieving complete stable remission, 40%−50% maintaining minimal manifestation status, and 10%−20% experiencing persistent moderate to severe symptoms (1, 98). Adverse prognostic indicators include thymoma, anti-striated muscle antibodies, rapid generalization, bulbar and respiratory muscle involvement, and delayed treatment (109, 110).

LOMG demonstrates the poorest prognosis, with only 15%−20% achieving complete stable remission, 50%−60% requiring sustained immunosuppression for maintenance control, and 20%−30% experiencing refractory disease (74, 99). Myasthenic crisis incidence (25%−35%) and mortality rates (8%−15%) significantly exceed those of younger patients, with increased intensive care requirements and delayed recovery (104, 105). Multiple comorbidities, reduced physiological reserve, and increased susceptibility to healthcare-associated infections collectively contribute to poor prognosis in elderly patients (111).

### 5.5 Central nervous system cellular senescence and senescence-associated secretory phenotype (SASP)

Beyond immunosenescence, cellular senescence of central nervous system (CNS) parenchymal cells may represent another critical mechanism linking aging to central symptoms in MG. Cellular senescence is a stable state of cell cycle arrest accompanied by distinctive phenotypic alterations, among which the acquisition of the senescence-associated secretory phenotype (SASP) is of greatest pathological significance (130, 131).

Within the CNS, microglia and astrocytes progressively accumulate senescent phenotypes with advancing age. Senescent microglia exhibit diminished phagocytic function and reduced immune surveillance capacity while simultaneously acquiring a pro-inflammatory phenotype (132, 133). Senescent astrocytes lose their normal neurosupportive functions, including glutamate uptake, ion homeostasis maintenance, and neurotrophic factor secretion (134). These senescent cells continuously secrete an array of pro-inflammatory cytokines (IL-6, IL-1β, and TNF-α), chemokines (CCL2, CXCL10), matrix metalloproteinases (MMP-3, MMP-9), and reactive oxygen species (ROS) through SASP, thereby creating a chronic pro-inflammatory microenvironment (130, 131, 135).

The potential mechanisms by which SASP contributes to LOMG pathophysiology include:

(1) Priming effect: senescent cells accumulated within the CNS create a chronic pro-inflammatory microenvironment through SASP, lowering the response threshold of brain parenchyma to peripheral immune signals. Studies have demonstrated that senescent cell accumulation is closely associated with enhanced neuroinflammatory reactivity (132, 136). This primed state may explain why LOMG patients exhibit more pronounced central responses to similar levels of peripheral inflammatory stimuli compared with younger patients.
(2) Inflammatory amplification effect: when peripheral MG-related inflammatory signals (such as elevated IL-6 and TNF-α) reach the CNS, SASP factors synergize with peripheral inflammatory mediators, resulting in inflammatory amplification. Chemokines within the SASP can also promote infiltration of peripheral immune cells into the CNS, further exacerbating neuroinflammation (135, 137).
(3) Impaired repair: matrix metalloproteinases within the SASP can degrade the extracellular matrix, compromising blood-brain barrier integrity (138). Concurrently, senescent glial cells lose their normal neuroprotective and reparative functions, inhibiting neuroregeneration and synaptic plasticity, and delaying injury repair processes (134, 139).

These mechanisms may explain why LOMG patients often exhibit more severe fatigue and cognitive impairment even when their peripheral disease activity is comparable to that of younger patients. Notably, senolytic drugs that selectively eliminate senescent cells (such as dasatinib plus quercetin and navitoclax) have demonstrated the potential to improve neurological function in preclinical studies across various age-related diseases (140, 141). Additionally, senomorphic strategies that suppress SASP (such as JAK inhibitors and NF-κB inhibitors) may attenuate the detrimental paracrine effects of senescent cells (142). These emerging therapeutic strategies may provide novel treatment approaches for central symptoms in LOMG and warrant further investigation in future studies.

### 5.6 Potential therapeutic targets for neuroinflammation and age-related components

Conventional MG treatments primarily target peripheral autoimmune responses and include cholinesterase inhibitors, glucocorticoids, immunosuppressants, and biologics targeting B cells or complement. However, LOMG patients frequently present with non-muscular symptoms that respond poorly to conventional therapy, including chronic fatigue, cognitive dysfunction, and mood alterations. These symptoms may be closely associated with neuroinflammation and age-related cellular senescence (143, 144). Based on the aforementioned pathophysiological mechanisms, the following targeted strategies may provide novel approaches for comprehensive LOMG management.

#### 5.6.1 Microglial signal inhibitors

Microglia, as resident immune cells of the CNS, play a central role in neuroinflammation. Colony-stimulating factor 1 receptor (CSF1R) is a key regulator of microglial survival and proliferation. PLX5622 is a highly selective, blood-brain barrier-penetrant CSF1R inhibitor that has been shown to effectively deplete brain microglia (145). In multiple neurodegenerative disease models, PLX5622 improves cognitive function and neuropathological changes by eliminating chronically activated microglia and reducing NADPH oxidase 2 (NOX2)- and NOD-like receptor protein 3 (NLRP3) inflammasome-associated neuroinflammation (146, 147). Minocycline, another microglial inhibitor, suppresses microglial activation and pro-inflammatory cytokine release, demonstrating neuroprotective effects in various neuroinflammation models (148). These strategies may be particularly applicable to LOMG patients with cognitive impairment.

#### 5.6.2 JAK inhibitors

The Janus kinase-signal transducer and activator of transcription (JAK-STAT) signaling pathway mediates intracellular signal transduction of multiple pro-inflammatory cytokines (particularly IL-6) and plays a critical role in autoimmune diseases and neuroinflammation. Baricitinib and tofacitinib are JAK1/2 inhibitors approved by the United States Food and Drug Administration (FDA) for autoimmune diseases including rheumatoid arthritis (149, 150). Studies have demonstrated that baricitinib effectively inhibits the IL-6/JAK-STAT pathway, reduces T helper 1 (Th1) and Th17 cell differentiation, and decreases pro-inflammatory cytokine production (151). In the experimental autoimmune encephalomyelitis (EAE) model, baricitinib significantly attenuates clinical symptoms, delays disease onset, reduces demyelination and immune cell infiltration, and effectively decreases the numbers of microglia and astrocytes in the CNS (152). Notably, baricitinib can penetrate the blood-brain barrier and has shown potential to reduce neuroinflammation in models of human immunodeficiency virus (HIV)-associated neurocognitive disorders (152). For MG patients presenting with elevated serum IL-6 levels, JAK inhibitors may provide additional therapeutic benefits.

#### 5.6.3 NLRP3 inflammasome inhibitors

The NLRP3 inflammasome is a critical component of the innate immune system. Its activation leads to caspase-1 activation and maturation and release of interleukin-1β (IL-1β) and IL-18, contributing to the pathological processes of multiple neurodegenerative diseases (153). MCC950 is a potent, highly selective NLRP3 inhibitor that blocks NLRP3 activation at nanomolar concentrations without affecting the absent in melanoma 2 (AIM2), NLR family CARD domain-containing protein 4 (NLRC4), or NLRP1 inflammasomes (154). In the Alzheimer's disease APP/PS1 transgenic mouse model, MCC950 inhibits microglial activation, promotes amyloid-β (Aβ) clearance, and improves cognitive function (155). In cerebral ischemia-reperfusion models, MCC950 not only attenuates neuronal pyroptosis but also inhibits ferroptosis (156). For patients with severe MG accompanied by systemic inflammatory responses, NLRP3 inhibition may represent a promising adjunctive therapeutic strategy.

#### 5.6.4 Senolytic drugs

Senescent cells continuously release pro-inflammatory factors through SASP, creating a chronic inflammatory microenvironment. Senolytic drugs selectively eliminate these detrimental cells by targeting anti-apoptotic pathways in senescent cells. The combination of dasatinib (a tyrosine kinase inhibitor) plus quercetin (a natural flavonoid compound) represents the most extensively studied senolytic regimen (141). In aged mouse models, D+Q treatment reduces senescent cell burden in both peripheral tissues and the CNS, improves cognitive function, and reduces microglial activation (157). In tauopathy mouse models, D+Q treatment protects blood-brain barrier integrity, improves cerebral oxygen metabolism, and attenuates brain atrophy and tau hyperphosphorylation (158). Multiple clinical trials are currently evaluating the safety and efficacy of D+Q in patients with mild cognitive impairment and Alzheimer's disease (159). Navitoclax (ABT-263) is another senolytic drug that functions by inhibiting B-cell lymphoma 2 (Bcl-2) family proteins. For LOMG patients with chronic fatigue, senolytic strategies may alleviate SASP-mediated neuroinflammation by eliminating senescent glial cells in the CNS.

#### 5.6.5 Mitochondrial protectants

Mitochondrial dysfunction is a common feature of neurodegeneration and cellular senescence, leading to reduced adenosine triphosphate (ATP) generation, excessive ROS production, and apoptosis. Elamipretide (SS-31) is a mitochondria-targeted tetrapeptide that selectively binds to cardiolipin in the inner mitochondrial membrane, stabilizes mitochondrial cristae structure, enhances electron transport chain efficiency, and reduces ROS production (160, 161). Preclinical studies have demonstrated that elamipretide enhances mitochondrial respiration, promotes mitochondrial biogenesis, inhibits neuroinflammation (TNF-α, IL-6, and NLRP3 pathways), reduces toxic protein accumulation, and prevents neuronal apoptosis (162). MitoQ is another mitochondria-targeted antioxidant that reduces Aβ accumulation and oxidative stress in Alzheimer's disease models (162). For MG patients presenting with manifestations of energy metabolism impairment (such as severe fatigue), mitochondrial protection strategies warrant exploration.

#### 5.6.6 Nrf2 agonists

Nuclear factor erythroid 2-related factor 2 (Nrf2) is the master regulator of cellular antioxidant defense, controlling the expression of multiple antioxidant and detoxification enzymes. Dimethyl fumarate is an FDA-approved Nrf2 agonist for multiple sclerosis treatment that activates the Nrf2/Kelch-like ECH-associated protein 1 (KEAP1) pathway, enhances cellular antioxidant capacity, and exerts immunomodulatory effects (163). In neuroinflammation models, Nrf2 activation reduces oxidative damage, protects neurons, and inhibits excessive microglial activation (164). For MG patients with oxidative stress-related symptoms, Nrf2 agonists may provide neuroprotective benefits. The main characteristics of these strategies are summarized in Table 3.

**Table 3:** Potential therapeutic strategies targeting neuroinflammation and age-related components in LOMG.

Strategy	Specific drugs/Approaches	Target and mechanism	Potential indications	References
Microglial signal inhibitors	PLX5622 (CSF1R inhibitor), Minocycline	Inhibit microglial activation and proliferation; reduce NOX2/NLRP3-associated neuroinflammation	LOMG with cognitive impairment	(145–148)
JAK inhibitors	Baricitinib, Tofacitinib	Block IL-6/JAK-STAT pathway; reduce Th1/Th17 differentiation	MG with elevated IL-6	(149–152)
NLRP3 inflammasome inhibitors	MCC950	Inhibit IL-1β/IL-18 maturation and release; attenuate pyroptosis and ferroptosis	Severe MG with systemic inflammation	(153–156)
Senolytic drugs	Dasatinib + Quercetin, Navitoclax (ABT-263)	Target anti-apoptotic pathways; eliminate SASP-producing senescent cells	LOMG with chronic fatigue	(141, 157–159)
Mitochondrial protectants	Elamipretide (SS-31), MitoQ	Stabilize mitochondrial cristae; reduce mtROS; stabilize mitochondrial membrane potential (ΔΨm)	MG with energy metabolism impairment	(160–162)
Nrf2 agonists	Dimethyl fumarate	Activate Nrf2/KEAP1 pathway; enhance antioxidant defense	Oxidative stress-related symptoms	(163, 164)

It should be emphasized that the above therapeutic strategies are currently based primarily on preclinical evidence and have not been systematically evaluated in MG patients. These targeted strategies may be particularly beneficial for improving non-muscular symptoms in LOMG patients (such as chronic fatigue and cognitive deficits), which often respond poorly to conventional immunosuppressive therapy. Well-designed clinical trials are urgently needed to evaluate the safety and efficacy of these novel interventions in specific MG subpopulations and to develop biomarkers predictive of treatment response.

### 5.7 Current research limitations and future directions

Although the mechanistic framework described above provides important perspectives for understanding age-related CNS pathology in LOMG, current research has significant limitations that need to be addressed in future studies.

#### 5.7.1 Methodological limitations

(1) Difficulty in establishing causality: current studies cannot distinguish between primary peripherally driven neuroinflammation and secondary neurodegenerative changes resulting from chronic disease. Whether the CNS abnormalities observed in MG patients represent direct consequences of the disease, adverse effects of treatment, or age-related comorbidities remains undetermined (165, 166). (2) Lack of direct human evidence: direct evidence of CNS pathology in MG patients is derived primarily from animal models (such as EAMG), with human autopsy studies being extremely rare. The limited existing autopsy reports have small sample sizes and often involve cases with concurrent neurological diseases, making it difficult to draw MG-specific conclusions (167). (3) Predominance of cross-sectional studies**:** longitudinal studies tracking the dynamic relationship between peripheral immune status and CNS changes are currently lacking. Cross-sectional designs cannot reveal the temporal evolution of peripheral-central interactions during disease progression, nor can they assess the long-term effects of therapeutic interventions on the CNS (168). (4) Confounding factors: LOMG patients often have multiple age-related comorbidities (such as hypertension, diabetes mellitus, and atherosclerosis) and are frequently on multiple medications. These factors themselves can affect CNS function and inflammatory status, making it difficult to isolate MG-specific CNS effects (169).

#### 5.7.2 Urgent research directions

(1) Longitudinal neuroimaging biomarkers: establishing a multimodal neuroimaging research framework is essential for elucidating CNS involvement in MG. Specific techniques include: (i) combined PET-MRI imaging: using translocator protein (TSPO) ligands (such as [^11^C]PK11195 or [^18^F]DPA-714) to assess microglial activation status (170); (ii) diffusion tensor imaging (DTI)**:** monitoring white matter microstructural integrity changes, enabling detection of early axonal injury (171); (iii) dynamic contrast-enhanced MRI (DCE-MRI): quantifying dynamic changes in blood-brain barrier permeability (172); (iv) magnetic resonance spectroscopy (MRS): detecting alterations in brain metabolites (such as N-acetylaspartate [NAA], myo-inositol, and glutamate), reflecting neuronal injury and glial activation status (173). (2) Liquid biopsy biomarkers: development of peripheral blood or cerebrospinal fluid biomarker panels reflecting CNS pathology is needed. Neuroinflammation markers should include: neurofilament light chain (NfL, reflecting axonal injury), glial fibrillary acidic protein (GFAP, reflecting astrocyte activation), and soluble triggering receptor expressed on myeloid cells 2 (sTREM2, reflecting microglial activation) (174, 175). Senescence markers should include: p16∧INK4a expression levels and SASP factor profiles [IL-6, MMP-3, plasminogen activator inhibitor-1 (PAI-1), etc.] (176). Combined detection of these markers may provide a non-invasive assessment tool for CNS involvement in MG. (3) Prospective cohort studies: establishing prospective cohorts of LOMG patients with baseline and regular follow-up data is essential, with systematic collection of the following information: (i) multimodal neuroimaging data (structural MRI, functional MRI, PET, DTI); (ii) comprehensive neuropsychological assessments (cognitive function, mood status, fatigue scales); (iii) peripheral immune phenotyping (T cell subsets, cytokine profiles, senescence markers); and (iv) treatment response and adverse event records. Such cohort studies will help elucidate the dynamic relationships between peripheral immune status and CNS changes, identify biomarkers predictive of CNS involvement risk, and provide a scientific basis for designing interventional clinical trials.

## 6 Conclusion

In summary, myasthenia gravis represents a clinically and immunologically heterogeneous disease, with its phenotype, disease course, diagnostic features, treatment responses, and prognosis exhibiting profound age dependence. This heterogeneity fundamentally arises from complex interactions among age-related immunosenescence, thymic involution, neuromuscular junction degenerative changes, and autoantibody profile evolution. With global population aging, the proportion of LOMG cases continues to rise, urgently necessitating deepened research into the following critical scientific questions: How do the molecular mechanisms of immunosenescence regulate MG onset and progression? The roles of chronic inflammatory states, T cell repertoire atrophy, and regulatory T cell dysfunction in MG across different age groups require elucidation. How do age-related degenerative changes of the neuromuscular junction influence susceptibility to autoantibody attack? Molecular mechanisms underlying presynaptic and postsynaptic structural and functional alterations and declining neuromuscular transmission safety factor warrant in-depth investigation. Why do anti-striated muscle antibodies increase significantly with age? Age-dependent mechanisms of muscle protein degradation, antigen exposure, and emergence of cross-reactive B cell clones merit exploration. How can optimized treatment strategies be developed for elderly MG patients? Considerations should include frailty assessment, polypharmacy management, individualized immunosuppression, and age-specific applications of novel targeted therapies.

Elucidating these age-dependent pathophysiological mechanisms, identifying biomarkers predictive of treatment responses across different age strata, and developing targeted interventions will constitute the core direction for future MG translational research and clinical practice advancement. The ultimate goal is to provide precise, individualized diagnostic and therapeutic protocols for MG patients across the entire lifespan.

## Glossary

ABCs, age-associated B cells; AD, Alzheimer's disease; ADCC, antibody-dependent cell-mediated cytotoxicity; AChE, acetylcholinesterase; AChR, acetylcholine receptor; AChR-Ab, acetylcholine receptor antibodies; AIRE, autoimmune regulator; Apaf-1, apoptotic protease activating factor-1; ATP, adenosine triphosphate; BBB, blood-brain barrier; BDNF, brain-derived neurotrophic factor; Blimp-1, B lymphocyte-induced maturation protein-1; CCL2, C-C motif chemokine ligand 2; CCL21, CC chemokine ligand 21; CCR7, CC chemokine receptor 7; CDC, complement-dependent cytotoxicity; CNS, central nervous system; CT, computed tomography; CTLA-4, cytotoxic T-lymphocyte-associated protein 4; CXCL10, C-X-C motif chemokine ligand 10; DAMPs, damage-associated molecular patterns; EOMG, early-onset myasthenia gravis; FcRn, neonatal Fc receptor; Foxp3, Forkhead box P3; GFAP, glial fibrillary acidic protein; GSDMD, gasdermin D; HIF-1α, hypoxia-inducible factor-1α; HLA, human leukocyte antigen; HMGB1, high mobility group box 1 protein; ICAM-1, intercellular adhesion molecule-1; IFN-γ, interferon-gamma; IGF-1, insulin-like growth factor-1; IgG, immunoglobulin G; IgG4, immunoglobulin G4; IL-1β, Interleukin-1 beta; IL-4, interleukin-4; IL-5, interleukin-5; IL-6, interleukin-6; IL-8, Interleukin-8; IL-10, interleukin-10; IL-13, interleukin-13; IL-17, interleukin-17; IL-18, Interleukin-18; IL-21, interleukin-21; IL-22, interleukin-22; JAK-STAT, janus kinase-signal transducer and activator of transcription; LOMG, late-onset myasthenia gravis; LRP4, low-density lipoprotein receptor-related protein 4; LTD, long-term depression; LTP, long-term potentiation; MAC, membrane attack complex; MAPK, mitogen-activated protein kinase; MCU, mitochondrial calcium uniporter; MG, myasthenia gravis; MGTX, Thymectomy Trial in Non-thymomatous Myasthenia Gravis Patients Receiving Prednisone Therapy; MHC, major histocompatibility complex; MMPs, matrix metalloproteinases; mPTP, mitochondrial permeability transition pore; MRI, magnetic resonance imaging; mtDNA, mitochondrial DNA; mTECs, medullary thymic epithelial cells; MuSK, muscle-specific kinase; MuSK-Ab, muscle-specific kinase antibodies; NADPH, nicotinamide adenine dinucleotide phosphate; NCLX, sodium-calcium exchanger; NF-κB, nuclear factor-kappa B; NK, natural killer (cells); NLRP3, NOD-like receptor protein 3; NMJ, neuromuscular junction; Nrf2-ARE, nuclear factor erythroid 2-related factor 2-antioxidant response element; PD-1, programmed cell death protein 1; PRRs, pattern recognition receptors; PSD, postsynaptic density; PTX3, pentraxin 3; RAGE, receptor for advanced glycation end products; RNS, reactive nitrogen species; RNS, repetitive nerve stimulation; ROS, reactive oxygen species; RyR, ryanodine receptor; SASP, senescence-associated secretory phenotype; SFEMG, single-fiber electromyography; TAK1, transforming growth factor-beta-activated kinase 1; TCM, central memory T cells; TCR, T cell receptor; TEM, Effector memory T cells; Tfh, Follicular helper T cells; TGF-β, transforming growth factor-beta; Th1, T helper 1 (cells); Th2, T helper 2 (cells); Th17, T helper 17 (cells); TLRs, toll-like receptors; TNF-α, tumor necrosis factor-alpha; TREM1, Triggering receptor expressed on myeloid cells 1; Tregs, regulatory T cells; UPR, unfolded protein response; VCAM-1, vascular cell adhesion molecule-1; ΔΨm, mitochondrial membrane potential.
