# Supplementary material for: Neuroinflammation: a critical bridge linking peripheral pathology and age-related degeneration in myasthenia gravis
Source: Front Med (Lausanne). 2026 May 1;13:1746161. doi: 10.3389/fmed.2026.1746161 (PMC13175833; doi:10.3389/fmed.2026.1746161)
Supplement: Supplementary file 1 [file Supplementary_file_1.docx]

**Supplementary Material 1**

**Literature Search Strategy**

**Search strategy:** A systematic literature search was conducted for this review. The following databases were searched: PubMed, Web of Science, Scopus, and the Cochrane Library. The search period spanned from January 2000 to May 2025. The following search term combinations were used: (1) ("myasthenia gravis") AND ("neuroinflammation" OR "microglia" OR "astrocyte" OR "blood-brain barrier"); (2) ("myasthenia gravis") AND ("aging" OR "immunosenescence" OR "late-onset"); (3) ("neuromuscular junction") AND ("degeneration" OR "aging"); (4) ("autoimmune neuromuscular disease") AND ("oxidative stress" OR "mitochondrial dysfunction").

**Age stratification definitions and inclusion/exclusion criteria:** The following age stratification criteria were adopted in this review: juvenile MG (age at onset ≤18 years), early-onset MG (EOMG; age at onset 19–49 years), and late-onset MG (LOMG; age at onset ≥50 years). Inclusion criteria were as follows: (1) original research articles or systematic reviews; (2) studies addressing MG immune mechanisms, neuroinflammation, or age-related pathological changes; (3) publications in English; and (4) articles published in peer-reviewed journals. Exclusion criteria were as follows: (1) conference abstracts and case reports with fewer than 3 cases; (2) articles without direct relevance to MG; and (3) duplicate publications. Given the focus of this review on the interplay between neuroinflammation and age-related neurodegeneration, priority was given to LOMG-related studies. Age-related studies were required to meet the following additional criteria: (1) explicit reporting of patient age at onset or age-stratified analysis; and (2) inclusion of data on immunosenescence markers (e.g., T cell subsets, inflammatory cytokines) or indicators of neurodegeneration.

**Literature screening process:** The initial search yielded 1,247 articles. After removal of duplicates, 876 articles remained. Screening by title and abstract excluded 612 irrelevant articles, and the remaining 264 articles underwent full-text assessment. Ultimately, 114 articles were included, comprising 52 clinical studies, 34 animal model studies, and 28 review articles. By age stratification, 41 articles were related to LOMG, 28 to EOMG, and 45 were basic mechanism studies without explicit age stratification. Literature screening was performed independently by two reviewers, with disagreements resolved through discussion or consultation with a third reviewer.
